# The Evolution of Fungal Metabolic Pathways

**DOI:** 10.1371/journal.pgen.1004816

**Published:** 2014-12-04

**Authors:** Jennifer H. Wisecaver, Jason C. Slot, Antonis Rokas

**Affiliations:** 1 Department of Biological Sciences, Vanderbilt University, Nashville, Tennessee, United States of America; 2 Department of Plant Pathology, The Ohio State University, Columbus, Ohio, United States of America; University of California-Riverside, United States of America

## Abstract

Fungi contain a remarkable range of metabolic pathways, sometimes encoded by gene clusters, enabling them to digest most organic matter and synthesize an array of potent small molecules. Although metabolism is fundamental to the fungal lifestyle, we still know little about how major evolutionary processes, such as gene duplication (GD) and horizontal gene transfer (HGT), have interacted with clustered and non-clustered fungal metabolic pathways to give rise to this metabolic versatility. We examined the synteny and evolutionary history of 247,202 fungal genes encoding enzymes that catalyze 875 distinct metabolic reactions from 130 pathways in 208 diverse genomes. We found that gene clustering varied greatly with respect to metabolic category and lineage; for example, clustered genes in Saccharomycotina yeasts were overrepresented in nucleotide metabolism, whereas clustered genes in Pezizomycotina were more common in lipid and amino acid metabolism. The effects of both GD and HGT were more pronounced in clustered genes than in their non-clustered counterparts and were differentially distributed across fungal lineages; specifically, GD, which was an order of magnitude more abundant than HGT, was most frequently observed in Agaricomycetes, whereas HGT was much more prevalent in Pezizomycotina. The effect of HGT in some Pezizomycotina was particularly strong; for example, we identified 111 HGT events associated with the 15 *Aspergillus* genomes, which sharply contrasts with the 60 HGT events detected for the 48 genomes from the entire Saccharomycotina subphylum. Finally, the impact of GD within a metabolic category was typically consistent across all fungal lineages, whereas the impact of HGT was variable. These results indicate that GD is the dominant process underlying fungal metabolic diversity, whereas HGT is episodic and acts in a category- or lineage-specific manner. Both processes have a greater impact on clustered genes, suggesting that metabolic gene clusters represent hotspots for the generation of fungal metabolic diversity.

## Author Summary

Fungi are important primary decomposers of organic material as well as amazing chemical engineers, synthesizing a wide variety of natural products, some with potent toxic activities, including antibiotics and mycotoxins. In fungal genomes, the genes involved in these metabolic pathways can be physically linked on chromosomes, forming gene clusters. This extraordinary metabolic diversity is integral to the variety of ecological strategies that fungi employ, but we still know little about the evolutionary processes involved in its generation. To address this question, we analyzed 247,202 enzyme-encoding genes participating in hundreds of metabolic reactions from 208 diverse fungal genomes to examine how two major sources of gene innovation, namely gene duplication and horizontal gene transfer, have contributed to the evolution of clustered and non-clustered metabolic pathways. We discovered that gene duplication is the dominant and consistent driver of metabolic innovation across fungal lineages and metabolic categories; in contrast, horizontal gene transfer appears highly variable both across organisms and functions. The effects of both gene duplication and horizontal gene transfer were more pronounced in clustered genes than in their non-clustered counterparts suggesting that metabolic gene clusters are hotspots for the generation of fungal metabolic diversity.

## Introduction

As one of the primary decomposers of organic material in nature, fungal species catabolize a wide diversity of substrates [1], including cellulose and lignin, the two most abundant biopolymers on earth [2]. Fungi are also superb chemical engineers, capable of synthesizing a wide variety of metabolites, including amino acids, small peptides, pigments and other natural products with potent toxic activities, such as antibiotics and mycotoxins [3]–[6].

Fungal metabolites have historically been divided into primary, that is metabolites essential for growth and reproduction, and secondary, which include ecologically important metabolites not essential to cellular life [7], [8]. However, this distinction is arbitrary when applied to metabolic pathways rather than their products not only because the essentiality of a given pathway is species-specific [9] but also because the pathways that generate primary and secondary metabolites are not mutually exclusive [10], [11]. Perhaps more informatively, pathways can be divided into those shared by most organisms, which can be considered as belonging to general metabolism, and those specialized pathways that have evolved in response to the specific ecologies of certain lineages and, as a result, are more narrowly taxonomically distributed.

An intriguing feature of specialized metabolic pathways in fungi is that constituent genes are often physically linked on chromosomes forming what are known as gene clusters [12], [13]. Fungal metabolic gene clusters are distinct from the developmental gene clusters typically found in animal genomes, such as the Hox gene clusters; whereas animal gene clusters are composed of tandemly duplicated genes [14], [15], fungal metabolic gene clusters comprise genes that are evolutionarily unrelated. Fungal metabolic gene clusters participate in diverse activities including nitrogen [16], [17], carbohydrate [18], amino acid [19], and vitamin [12] metabolism as well as in xenobiotic catabolism [11], [20] and the biosynthesis of secondary metabolites [e.g.], [ 21]–[28].

Although this extraordinary metabolic diversity, whether in the form of clustered or non-clustered pathways, is integral to the entire spectrum of fungal ecological strategies (e.g., saprotrophic, pathogenic and symbiotic), we still know little about the evolutionary processes involved in its generation. Gene duplication (GD), a major source of gene innovation, is often implicated in the evolution of fungal metabolism [e.g.], [ 29]–[31], especially in the context of whole genome duplication (WGD) [32]–[34] and gene family expansion [35], [36]. Notable examples include the GD of enzymes involved in organic decay [30], starch catabolism [37], degradation of host tissues [31], [38], [39] and toxin production [36]. Repeated rounds of GD, followed by divergence and differential gene loss, have also been invoked to explain the evolution of the gene clusters that generate the diverse alkaloids produced by plant symbiotic fungi [4]. A second key source of metabolic gene innovation in fungi is horizontal gene transfer (HGT) [40]–[44]; significant cases include the transfer of genes involved in xenobiotic catabolism [45], [46], toxin production [45], [47], degradation of plant cell walls [48], [49], and wine fermentation [50]. More recently, HGT has been shown to be responsible for the transfer of entire metabolic gene clusters between unrelated fungi [11], [51]–[58].

Although both GD and HGT have been extensively studied in fungal genomes, how these two major sources of gene innovation have interacted with clustered and non-clustered metabolic pathways and sculpted their evolution is largely unknown. To address this question, we analyzed 247,202 enzyme-encoding genes from 208 diverse fungal genomes whose protein products participate in hundreds of metabolic reactions. We found that both GD and HGT were more pronounced in clustered genes than in their non-clustered counterparts. On average, 90.0% of clustered metabolic genes underwent GD and 4.8% underwent HGT, whereas 88.1% and 2.9% of non-clustered metabolic genes experienced GD and HGT, respectively. Remarkably, some genera appear to have undergone a larger number of HGT events than entire subphyla. While the effect of GD was largely stable across metabolic categories, HGT varied extensively. These results suggest that GD is the dominant and stable process underlying fungal metabolic diversity, whereas HGT's impact is more pronounced in specific lineages and metabolic categories. The disproportionate effect of GD and HGT on clustered genes renders metabolic gene clusters into hotspots of metabolic innovation and diversification in fungi.

## Results

### Clustered genes in fungi vary extensively across lineages and metabolic categories

**Figure 1:**
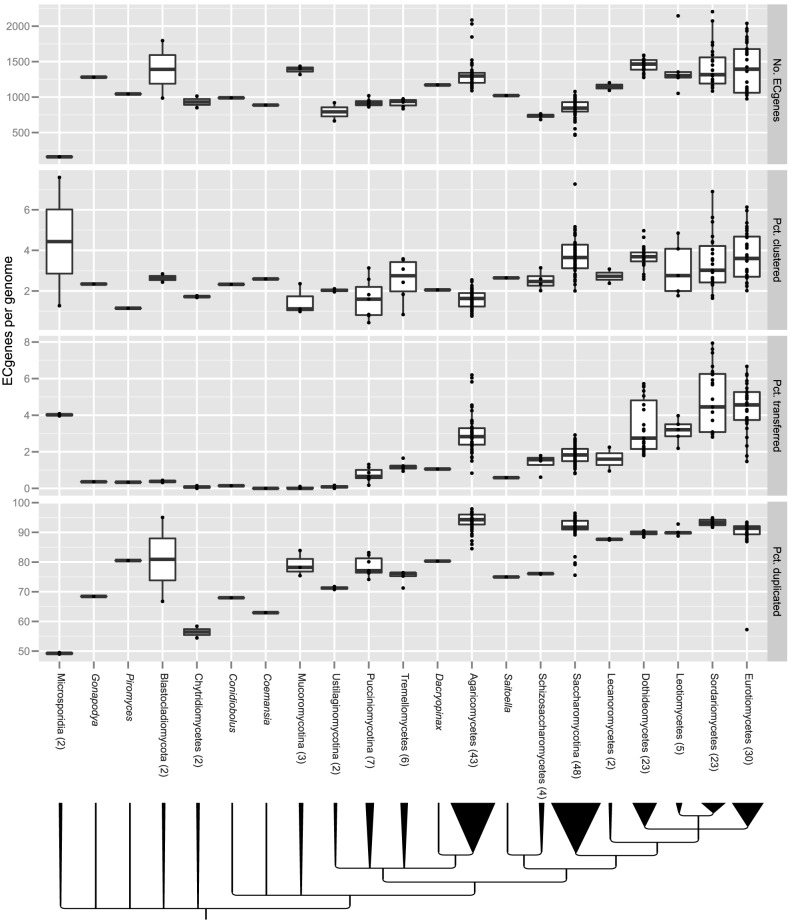
Variation in gene clustering, GD, and HGT across the fungal phylogeny. From top to bottom, the four box-and-whisker plots correspond to number of ECgenes per genome, percentage of clustered ECgenes per genome, percentage of horizontally transferred ECgenes per genome, and percentage of duplicated ECgenes per genome. The bottom and top of each box first and third quartiles (the 25th and 75th percentiles), respectively. The lower whisker extends from the box bottom to the lowest value within 1.5 * IQR (Inter-Quartile Range, defined as the distance between the first and third quartiles) of the first quartile. The upper whisker extends from the box top to the highest value that is within 1.5 * IQR of the third quartile. Data beyond the end of the whiskers are outliers and plotted as points. Numbers in parentheses after the lineages' names indicate numbers of genomes in each lineage; the numbers of genomes used from each lineage are also reflected by the widths of their branch triangles on the fungal species phylogeny shown at the bottom of the figure.

Analysis of 208 fungal genomes identified 247,202 Enzyme Commission (EC)-annotated metabolic genes (ECgenes for short), which encoded proteins catalyzing 875 distinct enzymatic reactions in 130 metabolic pathways (Figure 1; Table S1; Table S2). The percentage of the fungal proteome dedicated to metabolism was 15.4% in Saccharomycotina, 12.6% in Pezizomycotina and 8.9% in Agaricomycetes (Table S3; Figure S1).

Examination of fungal metabolism for the presence of metabolic gene clusters revealed that 3.0% (7,409) of ECgenes belonged to 3,408 distinct gene clusters, with the average genome containing 16.7 metabolic gene clusters and 36.3 clustered ECgenes (Table S3). The percentage of clustered ECgenes was highly variable across the major lineages, being more than two-fold greater in the two Ascomycota lineages, namely Pezizomycotina (3.6% of ECgenes) and Saccharomycotina (3.7%), than in Agaricomycetes (1.6%) (Figure 1, Table S3). For example, the plant pathogen *Fusarium solani* species complex species 11 (a.k.a., *Nectria haematococca*, Sordariomycetes) had 152 clustered ECgenes (representing 6.2% of its ECgenes), the most of any genome analyzed, the yeast *Torulaspora delbrueckii* (Saccharomycotina) had 59 clustered ECgenes (7.3%), whereas the ectomycorrhizal fungus *Laccaria bicolor* (Agaricomycetes) had only 14 clustered ECgenes (1.1%).

**Figure 2:**
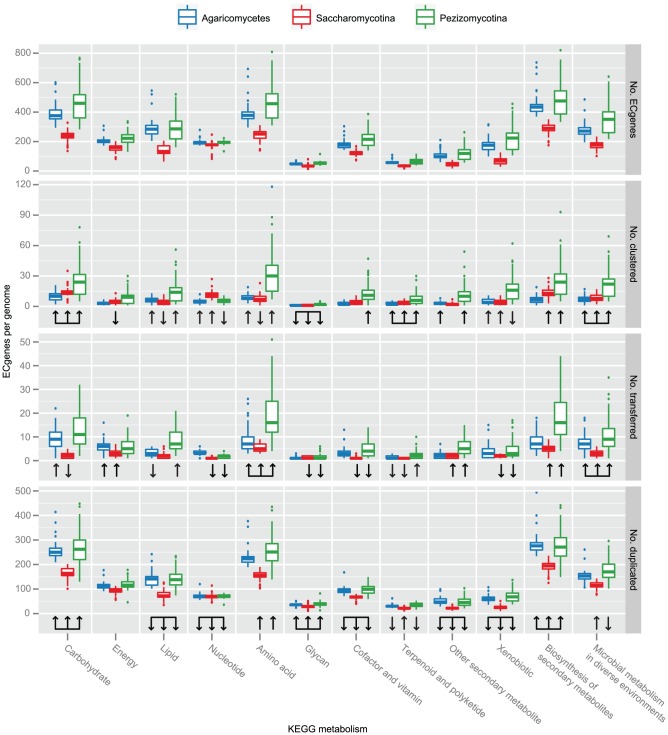
Over/underrepresentation of KEGG metabolic categories across three major fungal lineages. From top to bottom, the box-and-whisker plots correspond to number ECgenes per genome, number of clustered ECgenes per genome, number of transferred ECgenes per genome, and number of duplicated genes per genome. Agaricomycetes boxes are colored blue, Saccharomycotina boxes are colored red, and Pezizomycotina boxes green. Box-and-whisker convention is as described in Figure 1. Up arrows under boxes indicate overrepresentation, and down arrows indicate underrepresentation of the corresponding metabolic category in the corresponding lineage. Significance of differential representation was estimated using a two-tailed Fisher's exact test using a Benjamini & Hochberg adjusted *P* value≤0.05 to account for multiple testing (Table S4).

To test whether clustering was variable across fungal metabolism, we used the Kyoto Encyclopedia of Genes and Genomes (KEGG) metabolism hierarchy [10] to assign all ECgenes to 12 overlapping, higher-order metabolic categories (carbohydrate, energy, lipid, nucleotide, amino acid, glycan, cofactor/vitamin, terpenoid/polyketide, other secondary metabolite, xenobiotics, biosynthesis of secondary metabolites, and microbial metabolism in diverse environments). We found that the proportion of clustered ECgenes varied significantly across metabolic categories (Figure 2, Table S4). For example, clustered ECgenes from all lineages were significantly overrepresented in the KEGG categories carbohydrate and terpenoid/polyketide and underrepresented in the glycan category. In addition, the proportion of clustered ECgenes in a given category often varied significantly between lineages. For example, clustered ECgenes in the nucleotide and xenobiotic categories were only significantly overrepresented in Saccharomycotina and Agaricomycetes; clustered ECgenes in the same categories were underrepresented in Pezizomycotina (Figure 2). Similarly, clustered ECgenes in the amino acid and lipid categories were underrepresented in Saccharomycotina, whereas clustered ECgenes in these same categories were overrepresented in Pezizomycotina and Agaricomycetes (Figure 2).

### GD and HGT are differentially distributed across fungal lineages

To evaluate the impact of GD and HGT on fungal metabolism, we inferred GD and HGT events by reconciling the gene tree of each ECgene to the fungal species phylogeny [59]–[61]. Specifically, we assigned costs to GD, HGT, gene loss, and incomplete lineage sorting (ILS) and determined the most parsimonious combination of these four events to explain the ECgene tree topology given the consensus species phylogeny. Therefore, HGT events were inferred only when an ECgene tree topology was contradictory to the species phylogeny and could not be more parsimoniously reconciled using a combination of differential GD and gene loss. We evaluated multiple HGT costs and ultimately implemented a cost four times greater than the GD cost because it was the lowest HGT cost that recovered three published cases of HGT without any additional (e.g., potentially spurious) cases of HGT in the corresponding ECs (Table S5).

On average, 88.7% of ECgenes per genome were inferred to have undergone one or more GD events (Table S3). This percentage was lower in early diverging lineages; this was the case for both taxa with typical gene densities (e.g., Chytridiomycetes) as well as for the extremely reduced microsporidians, which displayed the lowest percentages of duplicated metabolic genes (49.0% and 49.5% of ECgenes in *E. cuniculi* and *E. intestinalis*, respectively). While the low percentages of GD in microsporidians are likely explained by genome streamlining, the low percentages observed in other early diverging lineages are harder to explain, although we note that their current sparse representation in the set of sequenced fungal genomes increases the uncertainty associated with estimating GD and HGT. In contrast, 93.7% of ECgenes underwent GD in the Agaricomycetes (Figure 1), with the button mushroom, *Agaricus bisporus*, having 97.0% of its ECgenes affected by GD (704 to 722 ECgenes depending on the strain). GD percentage was also high in the Saccharomycotina (91.4%; Figure 1), including in species belonging to the *Saccharomyces sensu stricto* group, where the average increased to 95.3%, most likely as a consequence of an ancient whole genome duplication [33], [62].

**Figure 3:**
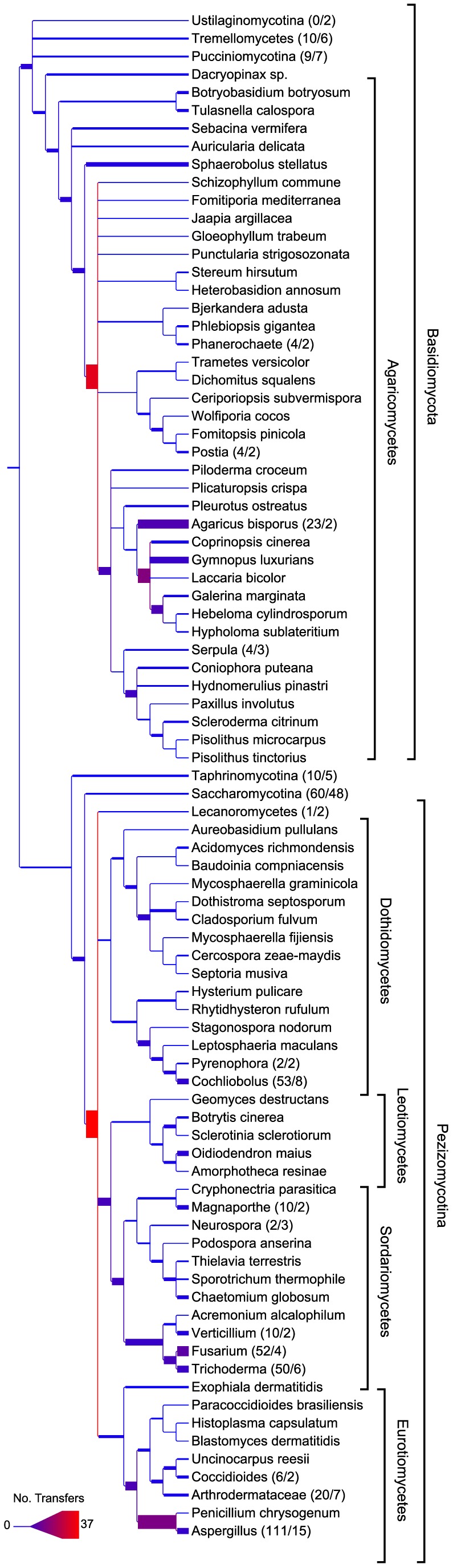
The episodic occurrence of HGT across the fungal species phylogeny. Numbers in parentheses indicate the number of HGT events and the number of genomes downstream of the collapsed nodes, respectively. Some clades have been collapsed for clarity; see Figure S2 for a depiction of the entire species phylogeny. The thickness and color of each branch corresponds to number of ECgenes transferred to each branch, adjusted by the number of genomes in the case of collapsed clades.

Our analysis also identified that on average 2.8% of ECgenes per genome had undergone one or more HGT events (Table S3), which could be traced back to 823 unique HGT events. The Pezizomycotina showed the highest percentage of HGT of all the major lineages, with an average 4.1% of ECgenes transferred per genome, and Saccharomycotina the lowest, with an average 1.8% of ECgenes transferred (Table S3; Figure 1). Remarkably, some Pezizomycotina genera showed nearly as many or more HGT events than the entire Saccharomycotina subphylum (Figure 3; Figure S2). For example, we identified 111 HGT events since the last common ancestor of the 15 *Aspergillus* species, the largest for any genus included in our analysis, but only 60 HGT events since the last common ancestor of the 48 Saccharomycotina genomes. Notwithstanding the fact that genome coverage and age are not the same across fungal genera, several other Pezizomycotina genera showed an abundance of HGT events including *Cochliobolus* (53 HGTs; 8 genomes), *Fusarium* (52 HGTs; 4 genomes), and *Trichoderma* (50 HGTs; 6 genomes). Within the Agaricomycetes, the highest concentration of HGT events was observed in the two *Agaricus bisporus* genomes (23 HGTs).

### GD and HGT rates are significantly higher for clustered genes in the Pezizomycotina

**Figure 4:**
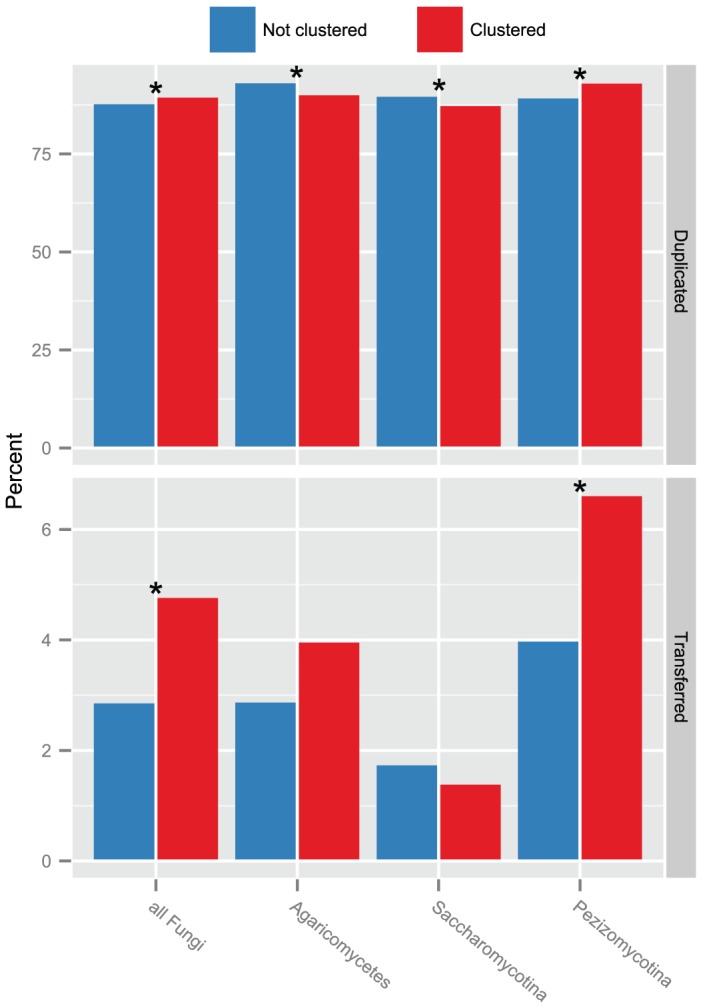
The association between gene innovation and gene clustering across three major fungal lineages. Percentage of non-clustered (blue bars) and clustered ECgenes (red bars) inferred to have undergone GD (top) and HGT (bottom). Asterisks (*) indicate statistically significant differences determined using a Benjamini & Hochberg adjusted *P* value≤0.05 in a two-tailed Fisher's exact test (Table S6).

Examination of the degree to which GD and HGT have differentially impacted clustered and non-clustered metabolic genes revealed significant differences (Figure 4; Table S6). On average, 90.0% of clustered ECgenes and 88.1% of non-clustered ECgenes underwent GD (*P* = 4.58×10^−4^). Similarly, 4.8% of clustered ECgenes underwent HGT compared to 2.9% of non-clustered ECgenes (*P* = 4.02×10^−12^). Examination of the impact of GD and HGT in the three major lineages shows that only in the Pezizomycotina was the percentage of GD and HGT significantly higher for clustered ECgenes than for non-clustered ECgenes (GD: 93.3% for clustered ECgenes versus 89.5% for non-clustered, *P* = 1.74×10^−11^; HGT: 6.6% for clustered ECgenes versus 4.0% for non-clustered, *P* = 2.77×10^−10^), suggesting that the trend is largely driven by Pezizomycotina. In fact, in both Saccharomycotina and Agaricomycetes GD was more common in non-clustered ECgenes than in clustered ECgenes (*P* = 0.02 and *P* = 0.01, respectively; Figure 4). HGT was more common in Saccharomycotina non-clustered ECgenes than in clustered ones, whereas in Agaricomycetes a higher incidence of HGT events was observed in clustered ECgenes, although neither of these associations was statistically significant (*P* = 0.54 and *P* = 0.16, respectively; Table S6).

### GD is consistent across fungal metabolism; HGT acts in a category- and lineage-specific manner

To test whether GD and HGT prevalence varied across fungal metabolism, we examined the rates of the two processes in each of the 12 KEGG metabolic categories across our three major lineages. We found that the effect of GD was generally consistent across metabolic categories, with 9/12 categories showing the same pattern of under/overrepresentation of duplicated ECgenes across the three lineages (Figure 2, Table S4). Specifically, the categories carbohydrate, glycan, and biosynthesis of secondary metabolites were overrepresented, the categories lipid, nucleotide, cofactor/vitamin, other secondary metabolites, and xenobiotics were underrepresented, whereas energy was not differentially represented in duplicated and non-duplicated ECgenes in all three lineages.

Unlike GD, HGT differentially affected metabolic categories in a lineage-specific fashion, with 10/12 categories differing in the pattern of under/overrepresentation of duplicated ECgenes across lineages (Figure 2, Table S4). For example, ECgenes in biosynthesis of secondary metabolites were overrepresented for HGT events in Pezizomycotina and Saccharomycotina, but not in Agaricomycetes. In contrast, ECgenes were overrepresented for HGT in lipid and terpenoid/polyketide in Agaricomycetes but underrepresented in the Pezizomycotina. Only 2 categories, amino acid and microbial metabolism in diverse environments, were overrepresented in transferred ECgenes across all three lineages.

## Discussion

Determining the relative role of GD and HGT with clustered and non-clustered metabolic pathways is important for understanding the evolution of the fungal metabolic repertoire. Examination of the synteny and evolutionary history of 247,202 ECgenes from 875 metabolic reactions across fungal diversity showed that GD is the dominant source of metabolic gene innovation in fungi, whereas HGT is variable across metabolic categories and fungal lineages. Both GD and HGT are more pronounced in clustered genes than in their non-clustered counterparts, suggesting that metabolic gene clusters can act as hotspots for the generation of fungal metabolic innovation.

### GD and HGT are sources of genetic novelty

On average 88.7% of fungal ECgenes retain the signature of one or more GD events in their ancestry compared to only 2.8% for HGT (Table S3). Even though these percentages are not directly comparable because reconciliation of ECgene histories with the species phylogeny requires that costs are assigned for every inferred GD or HGT event [60], our finding that nearly nine out of every ten metabolic genes have undergone GD suggests that this is the dominant source of gene innovation underlying fungal metabolism. These results are consistent with the hypothesis that specialized metabolic pathways evolve via GD from general metabolic precursors. Support for this hypothesis has come from phylogenetic analysis of single gene families [63], [64] such as the polykeytide synthases, which share a common evolutionary origin with the fatty acid synthases of general metabolism [65]. Further diversification of genes involved in specialized pathways may occur through additional duplication, functional divergence and differential loss in response to variable ecological pressures as has been proposed for polyketide, nonribosomal peptide and alkaloid biosynthesis genes [4], [66]–[68].

Our analysis showed that certain lineages in the Pezizomycotina and Agaricomycetes have increased HGT rates. Interestingly, bacteria-to-fungi HGT events are also elevated within Pezizomycotina, particularly in *Fusarium* and *Aspergillus* genomes [43]. HGT of entire chromosomes has been reported in *Fusarium* [69], [70], a genus in our analysis, which in addition to *Aspergillus*, *Cochliobolus* and *Magnaporthe*, appears not only receptive to HGT but also includes highly virulent plant and animal pathogens, ecological lifestyles associated with many known cases of HGT [11], [45], [47], [51], [69]–[71]. Similarly, mycoparasitism in the genus *Trichoderma* may also provide ecological opportunities for fungal-to-fungal HGT.

**Figure 5:**
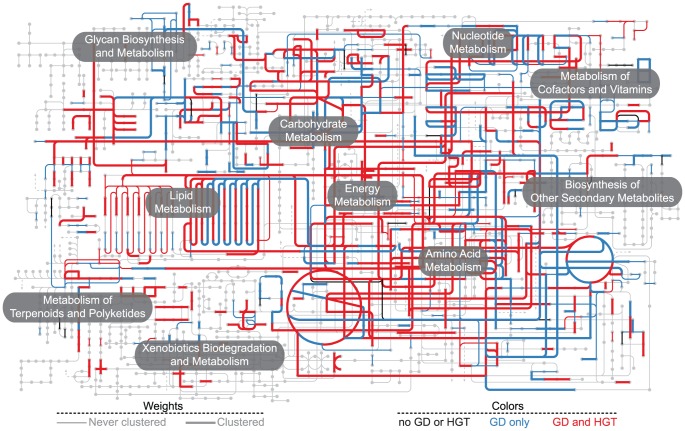
The fungal metabolic network of interactions between gene clustering and two major sources of gene innovation (GD and HGT). Nodes of the metabolic network correspond to KEGG compounds. Thick edges of the metabolic network correspond to EC numbers from clustered ECgenes in one or more fungal species, whereas thin edges to EC numbers whose genes show no history of gene clustering. Colored edges correspond to EC numbers whose ECgenes have undergone HGT *and* GD (red), GD (blue), or show no history of GD or HGT (black). Note that none of the EC numbers in our dataset were affected by HGT alone. Pathway map created using iPATH2.0 [95].

GD alone or in combination with HGT affected nearly every reaction in fungal metabolism (727, 95.7% of ECs that passed the phylogenomic analysis; Figure 5). The effect of both GD and HGT varied between metabolic categories, suggesting that some pathways may tolerate the introduction of new genes better than others. One possible explanation for this variation is that the metabolic networks associated with the different functional categories have different degrees of connectivity. Genes whose products make up large protein complexes or that have many interacting partners exhibit less variation in copy number [35], perhaps because unbalanced increases in gene dosage can lead to malformed protein complexes and a buildup of toxic intermediates in metabolic pathways [72]–[74], and might be less likely to undergo GD [75], [76] as well as HGT [77]. In addition to gene dosage effects, deleterious interactions between native and horizontally acquired proteins that function as parts of multi-protein complexes, and as a consequence have distinct co-evolutionary histories, are likely also important barriers to HGT [77], [78].

Another possible explanation is that the source of the variation of GD and HGT lies in the differing functions encoded by these metabolic categories. Gene innovation is often correlated with molecular function, with informational genes such as those involved in DNA replication, transcription and translation duplicated and transferred less often than metabolic genes [35], [76], [78]. Within metabolism, one might expect that widely distributed pathways involved in universal metabolic functions, such as oxidative phosphorylation and the citric acid cycle, are more likely to be functionally constrained and, as a consequence, less likely to tolerate GD or HGT of their constituent genes. In contrast, GD and HGT might be more advantageous for specialized metabolic pathways that are under strong selection in fluctuating environments [11].

33 EC reactions are associated with 332 ECgenes that are never duplicated or transferred in our analysis; 31 of these 33 reactions (93.9%) are also never clustered (Table S7a). For the majority of these ECs, the reason for the apparent lack of GD or HGT is because they are represented by only a few ECgenes in our analysis; therefore, their ECgene trees consist of few taxa with topologies in agreement with the consensus species phylogeny. For other EC reactions in this set, strong selection pressure to maintain a single, native gene copy could explain the lack of GD and HGT. Only three genes annotated with EC reaction numbers and which were never duplicated or transferred in our analysis were present in the *Saccharomyces cerevisiae* genome (YNL219C [2.4.1.259], YBR003W [2.5.1.83], and YPR184W [3.2.1.33]). When examined against the yeast phenotype and interaction data from the *Saccharomyces* Genome Database (http://www.yeastgenome.org), these three genes displayed a variety of phenotypes and all their null mutants were viable (Table S7b). Interestingly, overexpression of two of the ECgenes (YNL219C [2.4.1.259] and YBR003W [2.5.1.83]) resulted in reduced rate of vegetative growth in *S. cerevisiae* (Table S7b), suggesting that the acquisition of additional gene copies through GD or HGT could be disadvantageous. Furthermore, one *S. cerevisiae* ECgene, a glycosyltransferase (YNL219C [2.4.1.259]) involved in the biosynthesis of asparagine-linked glycans, has a very complex interaction network of 315 described physical and genetic interactions (Table S6a), which could serve as an additional barrier to GD and HGT.

### Gene clusters are hotspots for metabolic novelty

3.0% of fungal genes examined in our study lie within gene clusters. This is likely a conservative estimate because ECgene annotation is better for general rather than specialized metabolism. Although our analysis includes many specialized pathways (Table S2), such as biotin production (KEGG map00780), nitrate assimilation (map00910) and terpenoid backbone biosynthesis (map00900), and the fraction of enzymatic reactions encoded by clustered ECgenes is extensive (441 reactions, 50.4% of ECs; Figure 5), lineage-specific genes involved in specialized metabolic pathways are less likely to be included. In addition, fungal metabolic gene clusters are often identified through the presence of one or more conserved synthesis genes (e.g., genes encoding polyketide synthase or nonribosomal peptide synthase enzymes); proper demarcation of associated genes encoding modifying enzymes (e.g., oxidases and transferases) is challenging because they often lack functional annotation and are lineage-specific, leading to underestimates of gene cluster size.

Gene clustering in fungi is positively associated with both GD and HGT, but this pattern appears to be driven by Pezizomycotina ECgenes (Figure 4). Saccharomycotina ECgenes cluster more often than the global fungal average but are less often affected by HGT, whereas Agaricomycetes display the opposite trend; they experience more HGT but less gene clustering (Figure S3). GD affects nearly all ECgenes, and this large sample size undoubtedly contributes to the statistical significance of its association with gene clustering, even though the fold increase in the percentage of GD events observed in clustered versus non-clustered ECgenes is only 1.02. In contrast, the effect of HGT on clustered genes is 1.66 fold greater than its effect on non-clustered genes.

The uniqueness and wide distribution of fungal metabolic gene clusters has given rise to many models that attempt to explain their formation and maintenance [53], [79]–[83]. For example, the selfish gene cluster model proposes that HGT allows gene clusters to avoid being lost by facilitating colonization of new genomes [84], [85]. Although several instances of HGT of fungal gene clusters have been discovered in recent years [11], [51]–[58], clustered pathways are also more likely to be lost than non-clustered ones [53]. The small percentage of clustered genes affected by HGT in our analysis (4.8%), albeit larger than the background percentage of transferred un-clustered genes (2.9%), suggests that selfishness is unlikely to be the predominant mechanism driving gene cluster formation and maintenance in fungi. Nevertheless, the association between metabolic gene clusters and GD/HGT suggests that gene clustering can facilitate the duplication and transfer of entire metabolic pathways. This is consistent with the view that the barriers to gene innovation acting on gene clusters may be lower than those acting on single genes because the latter undergo GD or HGT in the absence of their functional partners.

## Materials and Methods

### Enzyme annotation

A custom enzyme classification pipeline assigned EC numbers to protein-coding genes from the genomes of 208 fungi and 9 stramenopiles (five oomycetes and four algal relatives), which were included in this analysis because of published reports of HGT between oomycetes and fungi [44]. Each gene was queried against a database of KEGG orthology (KO)-annotated proteins from 53 KEGG Organisms (Table S8) using ublast (http://drive5.com/usearch) with an accel setting of 0.7 and minimum identity cutoff of 0.3. A KO term was assigned to the query for ublast hits with greater than 80% sequence identity and no more than 10% difference in length. In cases where highly similar matches were not recovered, KO terms were assigned to query sequences with respect to the ublast hits showing the lowest e-values; all ublast hits that followed the first e-value increase of 10^−50^ or greater were excluded. EC numbers were assigned according to KO term (http://www.genome.jp/kegg-bin/get_htext?ko00001.keg).

### Detection of fungal metabolic gene clusters

Fungal proteomes were screened for metabolic gene clusters as described [81]. Briefly, two ECgenes were considered clustered if they were separated by no more than 6 intervening genes according to published annotation and their EC numbers were nearest neighbors in one or more KEGG pathways. Gene clusters were inferred by joining overlapping metabolic gene pair ranges that were separated by no more than 6 intervening genes; the cutoff of 6 intervening genes was determined empirically with reference to previous analyses of both primary [52], [53] and secondary [54] metabolism clusters.

### Phylogenetic reconstruction and gene tree-species phylogeny reconciliation

We constructed a draft fungal species phylogeny using protein sequences of the widely used DNA-directed RNA polymerase II subunit RPB2 marker, which were aligned with mafft using the E-INS-i strategy [86]. The resulting alignment was trimmed with trimal using the automated1 strategy [87], and the topology was inferred using maximum likelihood (ML) as implemented in raxml version 7.2.8 [88] using a PROTGAMMALGF substitution model and rapid bootstrapping (100 replications). Branches with bootstrap support less than 50 were collapsed using the Consense module in the phylip program [89]. The final bifurcating and consensus (multifurcating) species phylogenies (File S1) were constructed by making targeted corrections to the RPB2 topology based on published literature (Table S9).

ECgene trees were constructed using a custom phylogenomic pipeline (Figure S4). Guide trees were first constructed for each ECgene family with mafft using the scores of pairwise global alignments [86] and rooted with the notung rooting optimization algorithm using event parsimony. This distance-based guide tree and the consensus species phylogeny were used to delineate groups of homologs by aiming to maximize taxonomic diversity while minimizing the number of paralogs in each gene tree. The ECgene sequences from each one of these groups of homologs were then extracted in FASTA format for phylogenomic analysis. FASTA files of ECgenes with less than 4 or more than 1000 sequences were excluded. Sequences were aligned in mafft using the auto strategy selection [86]. Alignments were trimmed in trimal using the automated1 trimming strategy [87], and trimmed alignments shorter than 150 amino acid residues were discarded. Phylogenetic trees were constructed using fasttree [90] with a WAG+CAT amino acid model of substitution, 1000 resamples, four rounds of minimum-evolution subtree-prune-regraft moves (-spr 4), and the more exhaustive ML nearest-neighbor interchange option enabled (-mlacc 2 –slownni).

Gene tree-species phylogeny reconciliation was performed in notung using its duplication, transfer, loss and ILS aware parsimony-based algorithm [59]–[61], [91]. Ambiguity in the fungal species phylogeny and low branch support in ECgene trees were handled through a multi-step approach. First, ECgene tree branches with less than 0.90 SH-like local support were collapsed using treecollapsercl v4 (http://emmahodcroft.com/TreeCollapseCL.html). This collapsed ECgene tree was rooted and its polytomies resolved against the bifurcating species phylogeny. This resolved ECgene tree was then reconciled to the multifurcating, consensus species phylogeny using a duplication cost of 1.5, loss cost of 1 and ILS cost of 0. Transfer costs of 2, 4, 6, 8, 10 and 12 as well as the option to prune taxa not present in the gene tree from the species phylogeny were evaluated. A transfer cost of 6 with the prune option enabled best recovered published cases of HGT between fungi (Table S5). Percent GD and HGT were expressed over the 152,835 fungal ECgenes that passed this reconciliation pipeline. Because a single ancestral HGT event could be recorded in multiple ECgene trees, we defined unique HGT events as all cases where ECgenes assigned to the same EC number were inferred to have undergone HGT to/from the same recipient/donor nodes in the species phylogeny.

### Statistical analyses

Fisher's exact tests were performed using the R function fisher.test with a two-sided alternative hypothesis [92]. *P* values were adjusted for multiple comparisons using the R function p.adjust with the Benjamini & Hochberg (BH) method [93]. Box-and-whisker plots were created using the R plotting system ggplot2 [94].

## Supporting Information

Figure S1 Variation in gene clustering, HGT, and GD across fungal lineages, expanded version. From top to bottom, the four box-and-whisker plots correspond to number of ECgenes per genome, percentage of clustered ECgenes per genome, percentage of horizontally transferred ECgenes per genome, and percentage of duplicated ECgenes per genome. Box-and-whisker convention is as described in Figure 1. Numbers in parentheses after the lineages' names indicate numbers of genomes in each lineage; the numbers of genomes used from each lineage are also reflected by the widths of their branch triangles on the fungal species phylogeny shown at the bottom of the figure.
(PDF)

Figure S2 HGT across fungal species phylogeny, expanded version. Numbers above branches indicate number of HGT events predicted to have occurred onto each branch. The thickness and color of each branch corresponds to number of ECgenes transferred to each branch.
(PDF)

Figure S3 Incidence of gene clustering, GD and HGT mapped onto the global metabolism networks of Pezizomycotina, Saccharomycotina and Agaricomycetes. Nodes of the metabolic network correspond to KEGG compounds. Thick edges of the metabolic network correspond to EC numbers from clustered ECgenes in one or more fungal species, whereas thin edges to EC numbers whose genes show no history of gene clustering. Colored edges correspond to EC numbers whose ECgenes have undergone HGT *and* GD (red), GD only (blue), HGT only (green), or show no history of GD or HGT (black). Pathway maps created using iPATH2.0 [95].
(PDF)

Figure S4 Phylogenomics pipeline. A schematic diagram showing the functional components and data flow of the phylogenomics pipeline and gene tree-species phylogeny reconciliation.
(PDF)

Table S1 List of genomes used.
(XLSX)

Table S2 List of KEGG categories and pathways used.
(XLSX)

Table S3 Average gene clustering, GD and HGT per genome.
(XLSX)

Table S4 Fisher's exact tests for over/underrepresentation of KEGG metabolic categories in ECgene subsets.
(XLSX)

Table S5 Number of inferred HGT events in different iterations of the pipeline vs published literature.
(XLSX)

Table S6 Fisher's exact tests for association between sources of gene innovation (i.e., GD or HGT) and gene clustering.
(XLSX)

Table S7 Analysis of yeast phenotype and interaction data. a) List of EC reactions associated with genes that are never duplicated or transferred in the notung analysis with corresponding gene name in *S. cerevisiae* and number of protein interactions where available. b) Phenotype data from the *Saccharomyces* Genome Database.
(XLSX)

Table S8 List of KEGG organisms used for ECgene annotation.
(XLSX)

Table S9 Curation to species phylogeny with references.
(DOCX)

File S1 Species phylogeny in newick format. Tree 1: raxml best tree of RBP2. Tree 2: Consense majority rule phylogeny. Tree 3: Curated consensus species phylogeny. Tree 4: Curated bifurcating species phylogeny.
(DOCX)
