# Correction: The Evolution of Fungal Metabolic Pathways

**DOI:** 10.1371/journal.pgen.1005449

**Published:** 2015-09-17

**Authors:** Jennifer H. Wisecaver, Jason C. Slot, Antonis Rokas

Information is missing from the Acknowledgments section. The Acknowledgments should include the following text:

We thank Igor Grigoriev, Adrian Tsang, Andriy Sibirny, Antonio Logrieco, Burt Bluhm, Christian Kubicek, Dan Cullen, David Archer, Donald Nuss, Gillian Turgeon, Jason Stajich, Jennifer Wortman, Joey Spatafora, Johannes Hackstein, John Davis, Luis Corrochano, Mary Berbee, Nada Krasevec, Patricia van Kuyk, Paul Dyer, Peter Crittenden, Randy Berka, Ronald de Vries, Rytas Vilgalys, Santiago Torres Martínez, Sarah Watkinson, Scott Baker, Sharon Doty, Stephen Goodwin, Steven Singer, Terry Hazen, Thomas Jeffries, and Thomas Mock for providing access to unpublished genome data produced by the U.S. Department of Energy Joint Genome Institute, a DOE Office of Science User Facility, supported by the Office of Science of the U.S. Department of Energy under Contract No. DE-AC02-05CH11231.
