# Supplementary figures and images for: The Evolution of Fungal Metabolic Pathways
Source: PLoS Genet. 2014 Dec 4;10(12):e1004816. doi: 10.1371/journal.pgen.1004816 (PMC4256263; doi:10.1371/journal.pgen.1004816)

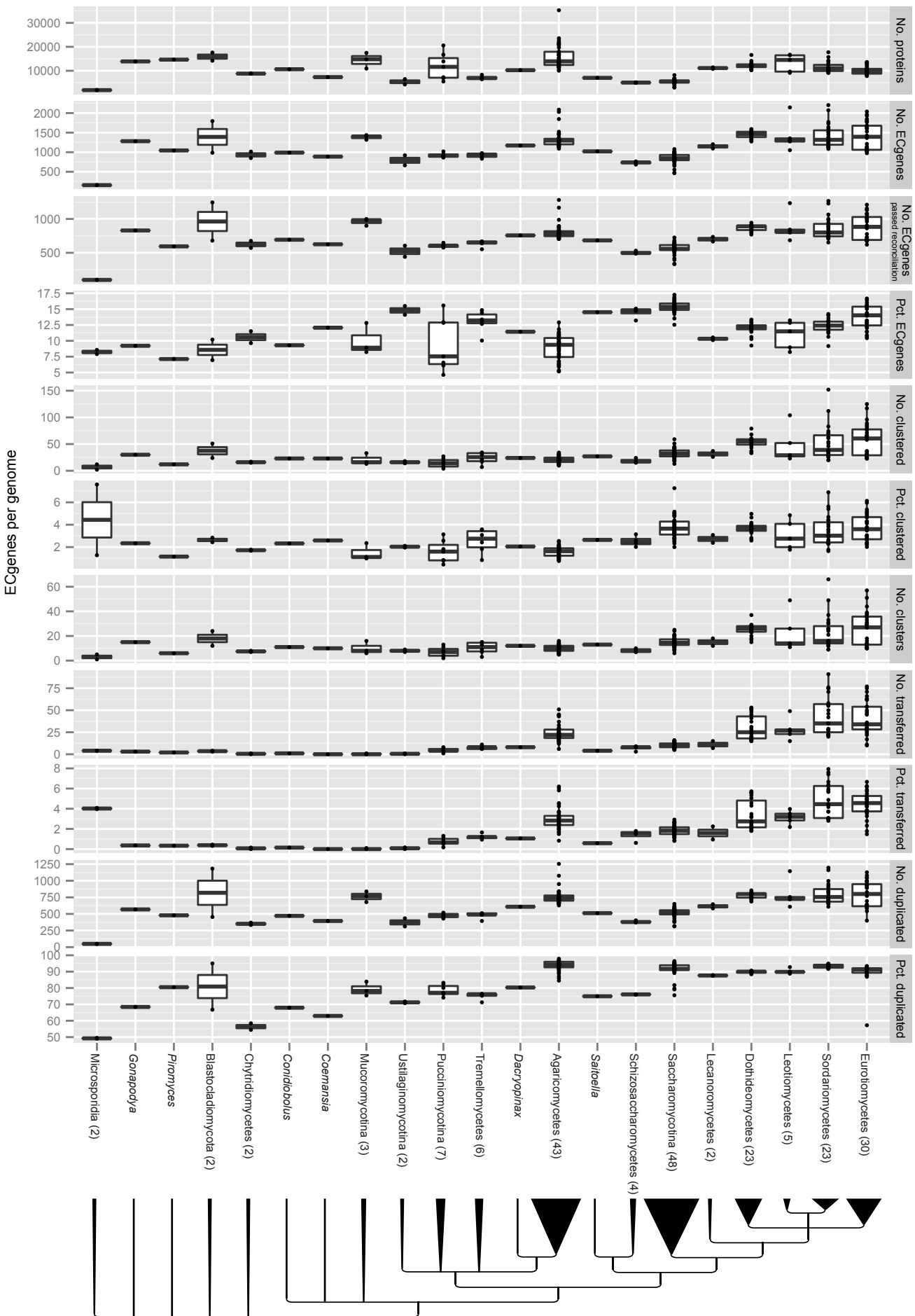

Supplement: Figure S1 — Variation in gene clustering, HGT, and GD across fungal lineages, expanded version. From top to bottom, the four box-and-whisker plots correspond to number of ECgenes per genome, percentage of clustered ECgenes per genome, percentage of horizontally transferred ECgenes per genome, and percentage of duplicated ECgenes per genome. Box-and-whisker convention is as described in Figure 1. Numbers in parentheses after the lineages' names indicate numbers of genomes in each lineage; the numbers of genomes used from each lineage are also reflected by the widths of their branch triangles on the fungal species phylogeny shown at the bottom of the figure. (PDF) [file pgen.1004816.s001.pdf]

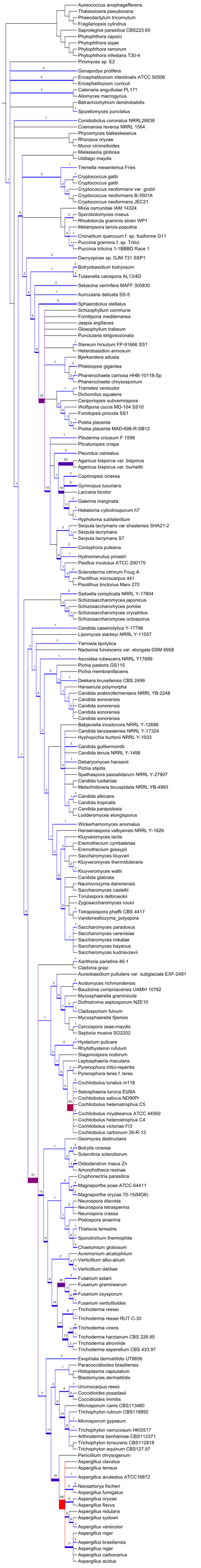

Supplement: Figure S2 — HGT across fungal species phylogeny, expanded version. Numbers above branches indicate number of HGT events predicted to have occurred onto each branch. The thickness and color of each branch corresponds to number of ECgenes transferred to each branch. (PDF) [file pgen.1004816.s002.pdf]

**For each gene cluster:**

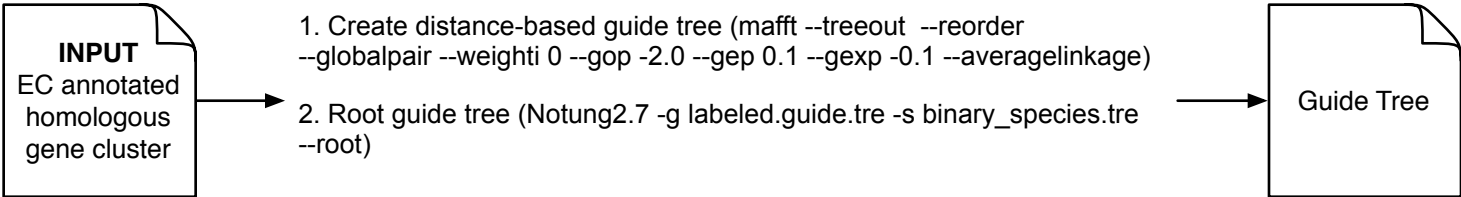

**For each ECgene:**

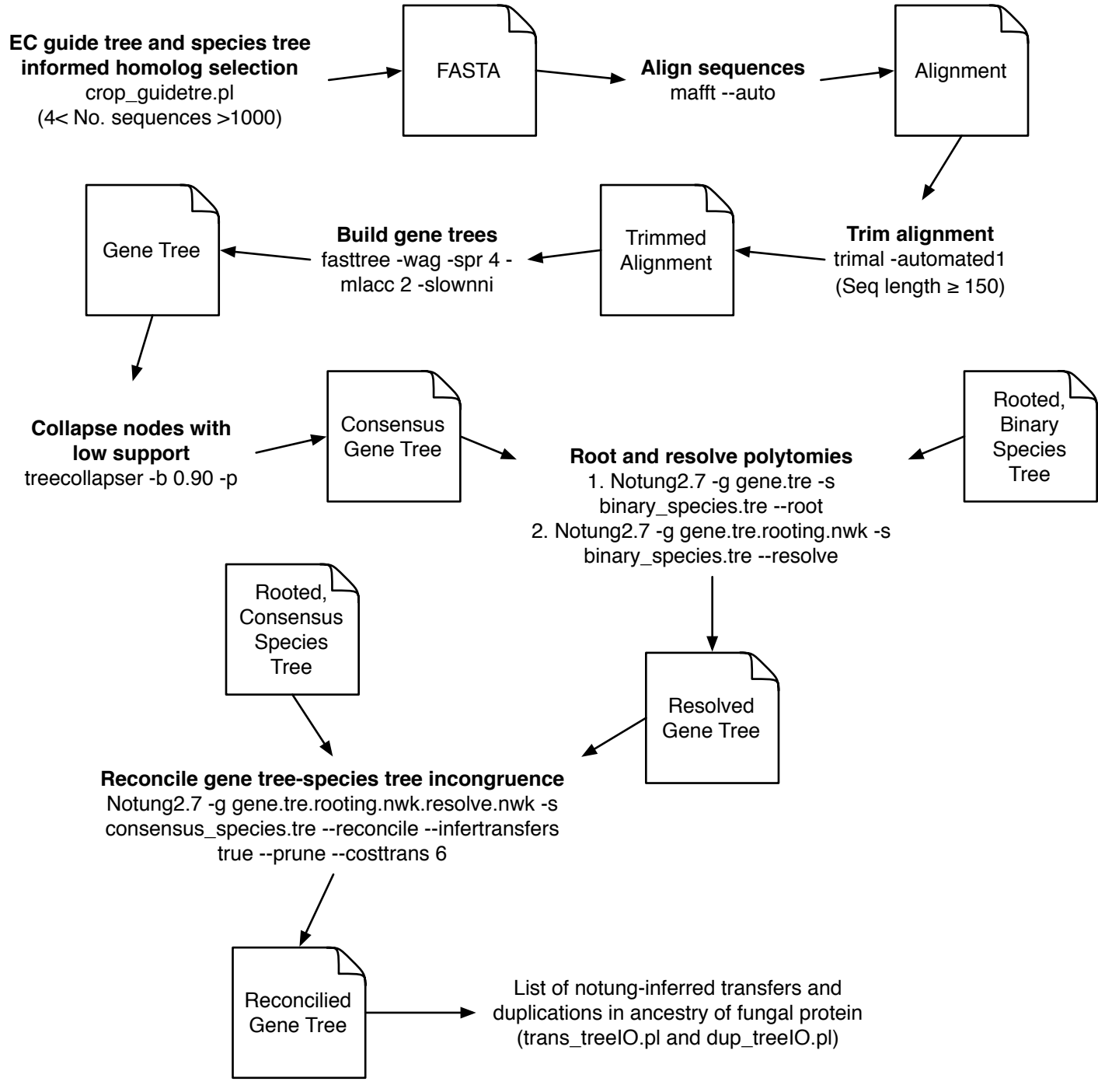

Supplement: Figure S4 — Phylogenomics pipeline. A schematic diagram showing the functional components and data flow of the phylogenomics pipeline and gene tree-species phylogeny reconciliation. (PDF) [file pgen.1004816.s004.pdf]
