# Supplementary material for: The Evolution of Fungal Metabolic Pathways
Source: PLoS Genet. 2014 Dec 4;10(12):e1004816. doi: 10.1371/journal.pgen.1004816 (PMC4256263; doi:10.1371/journal.pgen.1004816)

Agaricomycetes

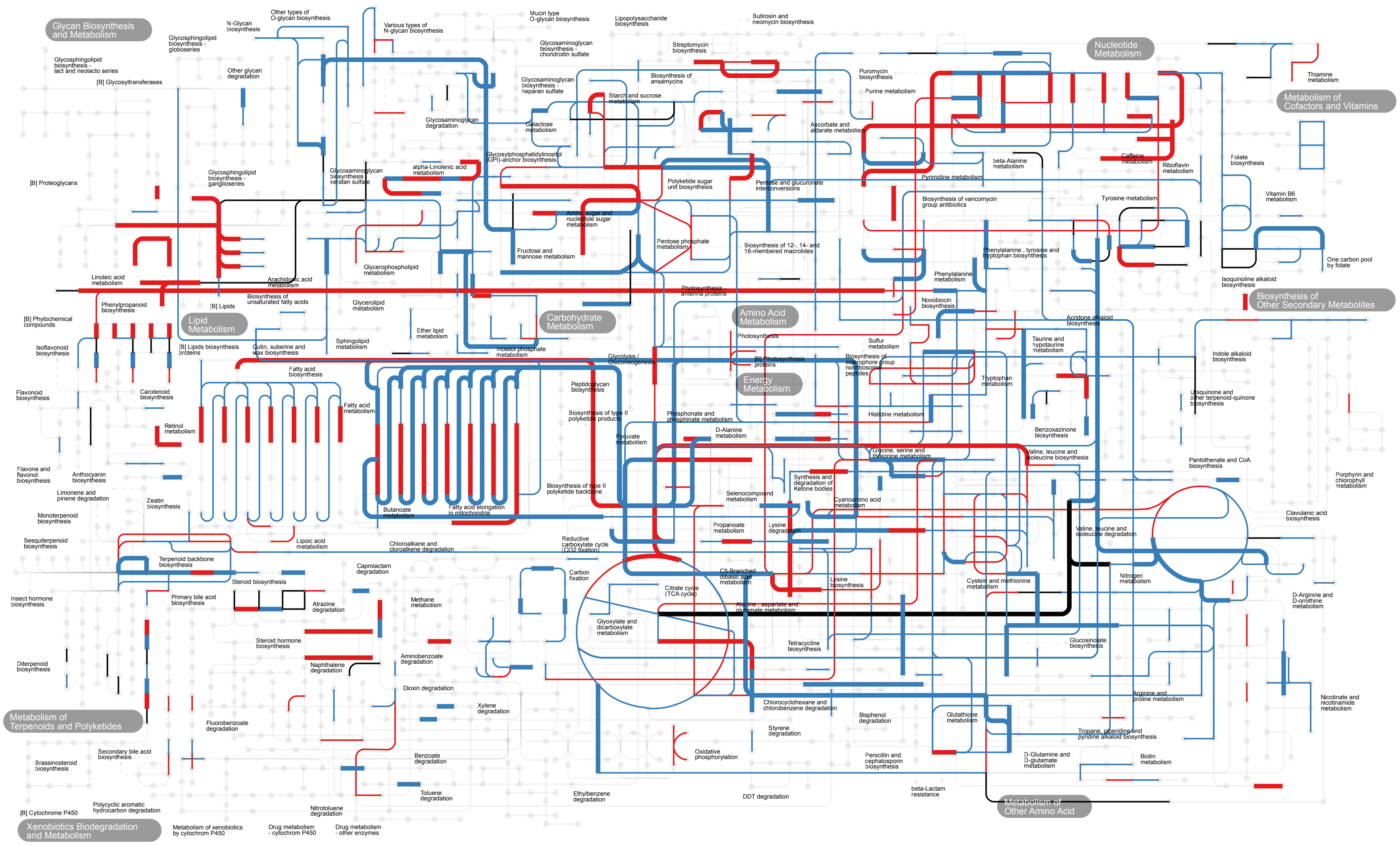

Weights

Non-clustered Clustered

Colors

no GD or HGT GD only GD and HGT HGT only

Pezizomycotina

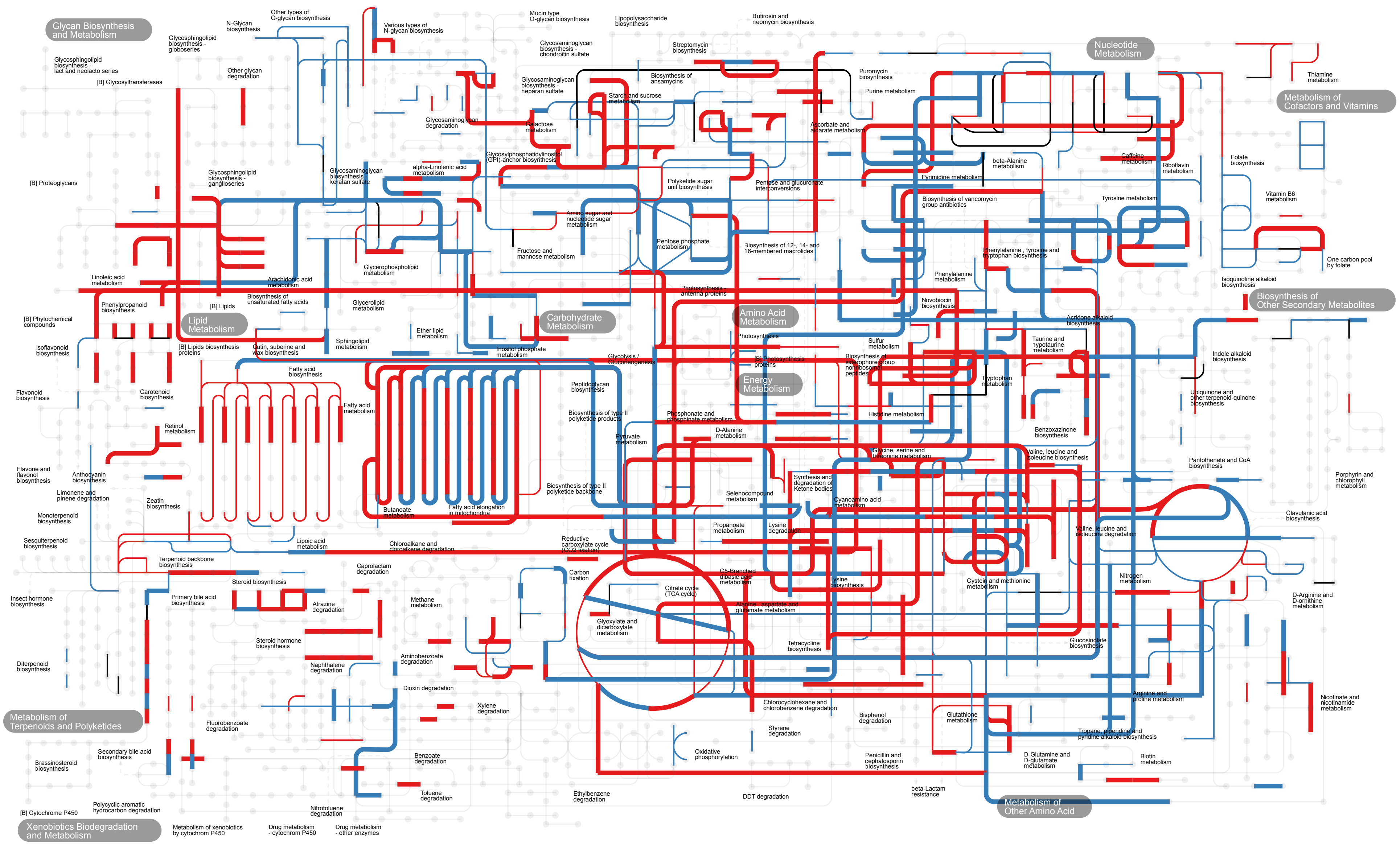

Weights

Non-clustered   Clustered

Colors

no GD or HGT   GD only   GD and HGT   HGT only

Saccharomycotina

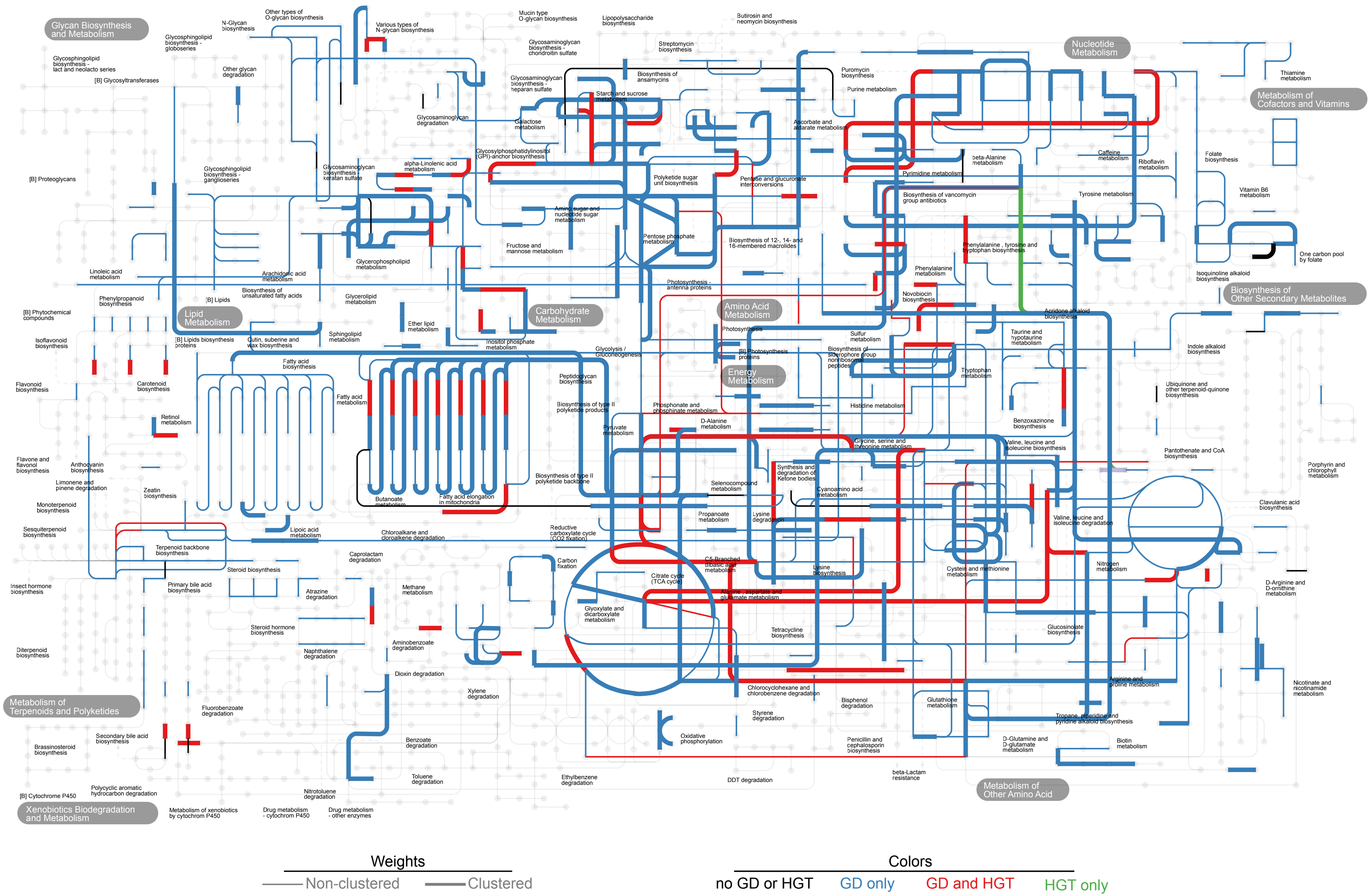

Supplement: Figure S3 — Incidence of gene clustering, GD and HGT mapped onto the global metabolism networks of Pezizomycotina, Saccharomycotina and Agaricomycetes. Nodes of the metabolic network correspond to KEGG compounds. Thick edges of the metabolic network correspond to EC numbers from clustered ECgenes in one or more fungal species, whereas thin edges to EC numbers whose genes show no history of gene clustering. Colored edges correspond to EC numbers whose ECgenes have undergone HGT and GD (red), GD only (blue), HGT only (green), or show no history of GD or HGT (black). Pathway maps created using iPATH2.0 [95]. (PDF) [file pgen.1004816.s003.pdf]
