# Supplementary material for: The Evolution of Fungal Metabolic Pathways
Source: PLoS Genet. 2014 Dec 4;10(12):e1004816. doi: 10.1371/journal.pgen.1004816 (PMC4256263; doi:10.1371/journal.pgen.1004816)
Supplement: Table S9 — Curation to species phylogeny with references. (DOCX) [file pgen.1004816.s013.docx]

Table S9: Curation to species phylogeny with references

| Corrections to RPB2 majority rule consensus phylogeny | Justification | Conflicts with RPB2 best tree |
| --- | --- | --- |
| Created *Fusarium* clade | Coleman et al., 2009 | yes |
| Created Xanpa1, Clagr2 clade | tolweb.org | yes |
| Created Rory, Mcir clade | Hoffmann et al., 2013 | yes |
| Added polytomies (Post,((Abibi,Abibi),(Ccin,Gymlu1,Lbic,(Galma1,(Hebcy1,Hypsu1))))) | Matheny et al., 2006 | yes |
| Created Cneb, Cnej clade | Findley et al., 2009 | no |
| Added polytomy (Trub,Mgyp,Tver,Aben,(Tton,Tequ)) | Bradshaw et al., 2013 | no |
| Collapsed Anigcbs51388, Aspbr1, Anig, Acar, Aspfo1 nodes | Gibbons & Rokas, 2013 | no |
| Collapsed Spoth2, Cglo node | Bradshaw et al., 2013 | no |
| Collapsed Ntet, Ncra node | Bradshaw et al., 2013 | no |
| Collapsed branching at base of Pezizomycotina | low support | no |
| Collapsed branching at base of Basidiomycota | low support | no |
| Collapsed Spar, Scer, Smik, Sbay, Skud nodes | Salichos & Rokas, 2013 | no |
| Collapsed *Candida sonorensis* node | low support | no |
| Collapsed Mcir, Pbla node | low support | no |
| Collapsed Settu, *Cochliobolus* nodes | low support | no |
| Collapsed Tdel, Zrou node | Kurtzman, 2011 | no |
| Collapsed Psti, Spapa node | low support | no |
| Collapsed Conco, Coere node | low support | no |
| Collapsed Mlar, Croqu node | low support | no |
| Collapsed Jaar1, Glotr1 node | Binder et al., 2005 | no |
| Collapsed branching near base of Agaricomycetes | Binder et al., 2005 | no |
| Collapsed Sebve1, Aurde1 node | Matheny et al., 2006 | no |
| Collapsed Agaricomycetes, Ustilaginomycotina node | Matheny et al., 2006 | no |
| Collapsed Dekbr2, Picme2 node | Kurtzman, 2011 | no |
| Collapsed Psti, Spapa node | Kurtzman, 2011 | no |
| Collapsed Canca1, Lipst1 node | Kurtzman, 2011 | no |
| Collapsed Ylip, Nadfu1 node | Kurtzman, 2011 | no |

**References**

Binder, M., Hibbett, D. S., Larsson, K. H., Larsson, E., Langer, E., & Langer, G. (2005). The phylogenetic distribution of resupinate forms across the major clades of mushroom‐forming fungi (Homobasidiomycetes). *Systematics and Biodiversity*, *3*(2), 113–157. doi:10.1017/S1477200005001623

Bradshaw, R. E., Slot, J. C., Moore, G. G., Chettri, P., de Wit, P. J. G. M., Ehrlich, K. C., et al. (2013). Fragmentation of an aflatoxin-like gene cluster in a forest pathogen. *New Phytologist*, *198*(2), 525–535. doi:10.1111/nph.12161

Coleman, J. J., Rounsley, S. D., Rodriguez-Carres, M., Kuo, A., Wasmann, C. C., Grimwood, J., et al. (2009). The genome of *Nectria haematococca*: contribution of supernumerary chromosomes to gene expansion. *PLOS Genetics*, *5*(8), e1000618. doi:10.1371/journal.pgen.1000618

Findley, K., Rodriguez-Carres, M., Metin, B., Kroiss, J., Fonseca, A., Vilgalys, R., & Heitman, J. (2009). Phylogeny and phenotypic characterization of pathogenic Cryptococcus species and closely related saprobic taxa in the Tremellales. *Eukaryotic Cell*, *8*(3), 353–361. doi:10.1128/EC.00373-08

Gibbons, J. G., & Rokas, A. (2013). The function and evolution of the Aspergillus genome. *TRENDS in Microbiology*, *21*(1), 14–22. doi:10.1016/j.tim.2012.09.005

Hoffmann, K., Pawłowska, J., Walther, G., Wrzosek, M., de Hoog, G. S., Benny, G. L., et al. (2013). The family structure of the Mucorales: a synoptic revision based on comprehensive multigene-genealogies. *Persoonia*, *30*, 57–76. doi:10.3767/003158513X666259

Kurtzman, C. P. (2011). Phylogeny of the ascomycetous yeasts and the renaming of Pichia anomala to Wickerhamomyces anomalus. *Antonie Van Leeuwenhoek International Journal of General and Molecular Microbiology*, *99*(1), 13–23. doi:10.1007/s10482-010-9505-6

Matheny, P. B., Curtis, J. M., Hofstetter, V., Aime, M. C., Moncalvo, J.-M., Ge, Z.-W., et al. (2006). Major clades of Agaricales: a multilocus phylogenetic overview. *Mycologia*, *98*, 982–995.

Salichos, L., & Rokas, A. (2013). Inferring ancient divergences requires genes with strong phylogenetic signals. *Nature*, *497*(7449), 327–. doi:10.1038/nature12130
