# Supplementary material for: The Evolution of Fungal Metabolic Pathways
Source: PLoS Genet. 2014 Dec 4;10(12):e1004816. doi: 10.1371/journal.pgen.1004816 (PMC4256263; doi:10.1371/journal.pgen.1004816)
Supplement: File S1 — Species phylogeny in newick format. Tree 1: raxml best tree of RBP2. Tree 2: Consense majority rule phylogeny. Tree 3: Curated consensus species phylogeny. Tree 4: Curated bifurcating species phylogeny. (DOCX) [file pgen.1004816.s014.docx]

**File S1 Species phylogeny in newick format.** Tree 1: raxml best tree of RBP2. Tree 2: Consense majority rule phylogeny. Tree 3: Curated consensus species phylogeny. Tree 4: Curated bifurcating species phylogeny.

[tree 1: RAxML best tree of RPB2] (CocheC5_72858:0.00000094020327838334,(((Cocvi1_94880:0.00000094020327838334,Cocca1_101489:0.00000094020327838334)26:0.00000094020327838334,CocheC4_140984:0.00000094020327838334)67:0.00000094020327838334,((Coclu2_65013:0.00307562936164287393,((((((Hyspu1_4193:0.02840239489751242297,Rhyru1_2106:0.09230395985747230636)100:0.09203283915807325410,((Aurpuvarsub1_6397:0.22154602132373119039,(((Clafu1_190720:0.03516229390138447125,Dotse1_42537:0.07725614610005070160)100:0.12573438454304106116,(((Sepmu1_148144:0.09573897939421820158,Cerzm1_65146:0.13593034701552877963)64:0.03667940110710340290,Mfij_58161:0.09726528402707740328)87:0.03621558408338836771,Mgra_84586:0.19254979725355900189)47:0.02799453566098667953)100:0.10927728598030166107,(Aciri1iso_149678:0.11810297243832604264,Bauco1_65631:0.16670629144341952976)100:0.14407461807374546958)100:0.11445499758720430183)100:0.20406209075644188289,(((Clagr2_26465:0.00000094020327838334,Cgra_26465:0.00000094020327838334)100:0.20282075045534855851,(Xanpa1_58045:0.38041571833701565142,((Eder_HMPREF1120_04530T0:1.08642001734734860996,(((Pbra_06854T0:0.02297905272238366658,(Bder_04692T0:0.01071503881991899519,Hcap_04441:0.00457523515277820543)100:0.01311195402425682777)100:0.02373048336751310342,((Mcan_02390T0:0.00426059147488490070,((Tequ_07670T0:0.00000094020327838334,Tton_06405T0:0.00152348753868293140)72:0.00152290484319659615,((Aben_06116:0.00000094020327838334,Trub_05742T0:0.00457043882127444647)21:0.00000094020327838334,(Tver_00102:0.00158707249927868559,Mgyp_07388T0:0.00158769992846093588)64:0.00159022532161518242)66:0.00000094020327838334)74:0.00652399225349126373)100:0.05250567011643079290,(Uree_02143:0.03532027893770009475,(Cpos_05757:0.00322418250290803891,Cimm_RS_v2_08123:0.00000094020327838334)100:0.00311118264883243579)100:0.03254120032570728682)88:0.02389811589586402288)95:0.03990417249920696213,(Pench1_67724:0.11638132576772917959,(Acla_065630:0.01915791404550918134,((Nfis_NFIA_114650:0.00427427057842428779,Afum_Afu7g01920:0.00501860614517672231)60:0.00378790160232501298,(Ater_01820:0.01161165780673377099,((((Acar_27118:0.00305455980829177085,Aspfo1_37284:0.00000094020327838334)62:0.00152517482472258928,(Anigcbs51388_317033268:0.00000094020327838334,(Aspbr1_35001:0.00458749277537172286,Anig_R317033268:0.00000094020327838334)16:0.00000094020327838334)49:0.00000094020327838334)53:0.00468333845520371109,(Aspac1_34713:0.02281225916036702314,(Afla_07897:0.00000094020327838334,Aory_AO090038000568:0.00000094020327838334)100:0.02214065102476721683)45:0.00766663034540289532)16:0.00430419166680137428,(Anid_AN9120:0.07961691298324016786,(Aspsy1_50074:0.00771795613815543825,Aspve1_47803:0.00141054229344297682)100:0.01430070700525072971)83:0.01245309616705252663)6:0.00513530972394154583)6:0.00766033266121377444)13:0.00613928671876301946)60:0.02226406925090907568)100:0.06267943822718304026)97:0.13118553913635525032)86:0.11599143407035479580,(((((Fgra_03041:0.07725981035899055771,(Fver_09301:0.00445573536035996035,Foxy_10639:0.00464199750480210477)100:0.04759991908909316088)100:0.07576269335552431039,(Nhae_70495:0.03832600142090112488,((TrireRUTC30_25448:0.00000094020327838334,Tree_79225:0.00000094020327838334)100:0.02173236547075686059,((Triha1_9591:0.00304225946168718659,Tvir_76818:0.00000094020327838334)80:0.00395980869187034009,(Trias1_77086:0.00459408213898034306,Tatr_151043:0.00000094020327838334)84:0.00847055384921068313)65:0.00655074955599800005)100:0.05008132037172971507)93:0.02822908088617691671)100:0.19402204385412535892,((Valb_04315T0:0.00000094020327838334,Vdah_05625T0:0.00295035758379388023)100:0.03649018450562460658,Acral1_1062944:0.06803858334914660022)100:0.12526942368594431998)98:0.06517633835488123584,((Cprs_93824:0.25753781907227335513,(Mory_04714T0:0.15353266379310562262,Mpoa_MAPG_02762T0:0.17535805816088961828)100:0.27345665368717309018)94:0.06462270274643711065,((Ndis_128789:0.00000094020327838334,(Ncra_01502:0.01326570067181013710,Ntet_118973:0.00148330664691573408)88:0.00305380763833888996)100:0.14099586539399042162,(Pans_PODANSg804:0.09796291988977150200,(Tter_74758:0.05474976255447280143,(Cglo_08507:0.10472587736371323508,Spoth2_2307570:0.04726563943204371843)94:0.02812795131546513491)98:0.04009054805004394484)100:0.06457279949088291782)100:0.20450138584897753491)97:0.05994187483568461788)100:0.30677351314915607405,(Gdes_03987T0:0.25587667723405621567,((Amore1_100240:0.01547819180200276507,Oidma1_38254:0.02734135205599598495)99:0.04092856015467788433,(Sscl_00885:0.01656751176069552051,Bcin_09004:0.01004365901846658755)100:0.11693677175444246175)92:0.03215172605139084150)95:0.04211246473093448878)96:0.07060633556484896545)69:0.02876040690377162923)57:0.04396657005085335274)86:0.07873091903581155848,(((Lipst1_73345:0.23677401895611396854,Canca1_32658:0.23838541816373329252)74:0.03126854171879333427,((Nadfu1_82421:0.15778897166578184641,Ylip_YALI0D03718g:0.20860849322862567989)100:0.07186192802103602562,(Ascru1_74872:0.54148153507168617082,((Ppas_254567802:0.18864951360950976689,(Hpol_50877:0.06857719135558366930,((Canar1_194128:0.05841643443498058946,(Cs941571_281:0.00000094020327838334,(Csonorensis_1007:0.00153282158109287843,(Cs918151_4380:0.00000094020327838334,Cs961314_4297:0.00000094020327838334)98:0.00000094020327838334)67:0.00153247824612146640)100:0.02055018766772305494)100:0.04135694828857558458,(Picme2_71110:0.09780747087589185262,Dekbr2_7243:0.12084219298130308151)55:0.03333740432400460890)44:0.02630018274985561691)100:0.32068564644622588045)97:0.08367437916581933754,((Wican1_53842:0.15175942487480340026,(Hanva1_56526:0.37363974740275618380,((((Spar_20881_YOR151C:0.00157914947567043377,(((Skud_AAT42141:0.03926076757499471354,Sbay_AAT42142:0.03037859037081962546)62:0.00889477879923279355,Smik_AAT42139:0.00000094020327838334)51:0.00533150860977744945,Scer_CAA99357:0.00000094020327838334)58:0.00471742739052793958)97:0.02238554159710492292,(Scas_AAT41619:0.02546593144373368242,Ndai_365984393:0.01456064270147137789)86:0.01745008300529775000)41:0.00372064568436284836,(Cgla138_50293059:0.04151164906208197614,((Vpol_186p2.t1:0.01965458915391586278,Tpha_367005628:0.04356616706031177233)98:0.02335862347429157326,(Zrou_ZYRO0D11770g:0.05400277687752273736,Tdel_367008806:0.01786695911326158931)74:0.01075672954612662616)52:0.00772495081076074973)19:0.00318993377328759611)100:0.12149183624206487397,(((Egos_AFR404C:0.02091778343622838210,Ecym_363752349:0.01128793383719217669)82:0.02710542525887668641,(Sklu_AAT12529:0.04673642648577091413,(Kwal_18783:0.01088375891164261415,Kthe_KLTH0G02970g:0.01104148100823476239)85:0.02773834152363281388)58:0.01170794612482386685)24:0.00927635414901681332,Klac_KLLA0B05577g:0.04328256089344882651)95:0.06550813636657483452)99:0.07612062433004043727)100:0.19568489844050407367)100:0.14980601548825284164,((Cgui_02833:0.05331066931083577182,(((Dhan_AAT12540:0.04539132026186536162,Cante1_117313:0.10217597654980063726)46:0.01526627063219154409,(((Clus_03629:0.03240819357690500474,Metbi1_32161:0.03841758513819894250)93:0.04295696310507684385,Hypbu1_152096:0.02580586153353276596)17:0.00141326990258211992,((Psti_34146:0.04131655016424549270,Spapa3_53113:0.04161654272372201230)85:0.01345712547047065756,((Ctro_04456:0.08225954452670988792,Calb_01218:0.07249871162555321380)100:0.05984371369805403795,(Cpar_00865:0.05512759271602120370,Lelo_01430:0.05302559002668764260)100:0.03646345342656667765)100:0.04062641336381508927)60:0.01079202758184529681)25:0.00748372676588678152)28:0.00866779466382834769,Canta1_315886:0.04242099497490824794)41:0.01421918833483084774)100:0.11788308993719588680,Babin1_159736:0.20522186411565587760)93:0.04646859170850735354)57:0.00871097113929979183)65:0.03177553627107636280)99:0.10661886595006239697)85:0.02885736324652359366)100:0.11224808412613658526,((Saico1_33216:0.71081027432960131041,(Sjap_04030:0.04077794399724105762,(Spom_SPAC23G3_01.1:0.01484028460835883426,(Soyt_03939T0:0.00391840293946714695,Soct_02073T0:0.01279344062162741415)100:0.04001118682521214581)99:0.04128807563551779858)100:0.22369004110709861388)81:0.06014214231345382106,((((Mixos1_14551:0.85760928385884038772,(((Putr_03658T0:0.28698503492624921618,Pgra_03411:0.02616448089868669835)100:0.03604123421174161568,(Mlar_47615:0.02430122247761712373,Croqu1_75937:0.02844707780194044577)62:0.01280962172248494534)100:0.25094997635729021646,(Sros_22879:0.09277947678474207649,Rhoba1_66967:0.10428498333965628697)100:0.26203745387087673535)97:0.06085969532384214592)100:0.12221757574429434334,((Dacsp1_23500:0.40779689255647305623,((Botbo1_27165:0.17661651009638973386,Tulca1_229733:0.30068729185177822671)74:0.04895839512458630138,((Sphst1_215952:0.15125453747341821575,(Fomme1_19006:0.40879063240014923419,((Hann_149541:0.05618568912713723523,Stehi1_89162:0.09349793395642429228)100:0.05991156726805797750,((((((Serlavarsha1_40234:0.00309907949755656997,(Slac_353774:0.00000094020327838334,SerlaS7_491686:0.00000094020327838334)100:0.00000094020327838334)100:0.02189641763885113654,(Conpu1_116067:0.06615553478619690120,(Hydpi2_174443:0.01097499213584098715,(Paxin1_64693:0.01823759761765172613,(Sclci1_1207496:0.02354731190650294001,(Pisti1_134697:0.01431562529359155927,Pismi1_671431:0.01223194931693229875)100:0.02200777244101390778)100:0.04039808507864883075)62:0.01335167361962312157)44:0.00751931043268944197)74:0.01413024114688769123)63:0.01149126481106246475,(Pilcr1_813588:0.02385854158911902487,(((Post_49642:0.09904598195250868820,(Abibu_117576:0.00000094020327838334,Abibi_113824:0.00319241560365471702)100:0.04504891646321916576)52:0.02036724285618238262,(Lbic_ws_229137:0.02306375833800951985,(Gymlu1_65950:0.08018056229310197014,(Hebcy1_452187:0.04679547771454699301,(Hypsu1_182098:0.04034423812086952721,Galma1_234313:0.03207915822795313898)37:0.01044178268071628210)90:0.02625199232897663937)1:0.00000094020327838334)17:0.01060856724765897596)25:0.00697133827139877638,Plicr1_171803:0.06640682200549022363)14:0.00757441435678372042)16:0.01356846711943886924)23:0.01731441858955379623,Ccin_01820:0.09191950742928775564)84:0.02473460606829199285,((Glotr1_135096:0.04689919845635943246,Jaaar1_121085:0.09467584325941810763)98:0.03398695141739246917,(Punst1_48856:0.14579769721406177219,((Bjead1_163844:0.08567832924662785454,(Phlgi1_125799:0.08329653393260544325,(Pchr_7545:0.01250475702221268311,Phaca1_247866:0.02251378255580232038)91:0.02590019967855433300)91:0.01421993335869875183)90:0.04021959962564613383,((Csub_110450:0.08706068597835361911,(Wolco1_135432:0.06391982483440947216,(Fompi1_100215:0.04928656283520800596,(PosplRSB12_1042494:0.00151132163640145924,Ppla_117322:0.00000094020327838334)100:0.04394426320727254365)89:0.01100711245524405024)92:0.01902529733185431837)86:0.01855698514943994806,(Dicsq1_164685:0.07998442520420924462,Trave1_33021:0.02931893639268204343)100:0.03162914823035176126)51:0.00393655703598443357)83:0.03256484835832020031)59:0.01509411375952903270)42:0.00847404836471306322)47:0.01941082719153453678,Scom_73589:0.32750183349037176317)29:0.01014422560522711819)43:0.01254253330531371335)87:0.02777410514833237795)75:0.04141986102090561284,(Aurde1_111514:0.12440006919821779141,Sebve1_331996:0.65057843223453293291)61:0.06037224241468836655)77:0.05881200063407586442)97:0.06575764108090335902)89:0.09262316118104017837,(Mglo_0889:0.20942171060689915296,Umay_AAS67525:0.26289275811510920411)100:0.34567065903270022265)66:0.06711587689321897587)47:0.04114205846412807149,(Tmes_70991:0.37824795069352429300,((Cneg_AEX32502:0.00000094020327838334,Cnew_AEX32502:0.00000094020327838334)58:0.00538873497647007500,((Cneb_CNBD2790.t1:0.00000094020327838334,Cnej_CND03540.t1:0.00000094020327838334)93:0.00282954893807966590,Cneo_H99_01235:0.00398000695151555072)10:0.00000094020327838334)100:0.28112006572114328629)100:0.60087273352680892202)66:0.05683013877149953319,((PirE2_17500:0.44589741222671069876,((Ganpr1_95064:0.92323742382642592919,((Catan1_53944:0.65362553957538793714,Amac_10446T0:0.29088582507079735473)100:0.34562628582677462630,(Eint_303390954:0.06089156349423031217,Ecun_0250_10:0.04585281344813358501)100:1.34920853337801260885)22:0.09597080061713500476)27:0.08752999988485135607,(((Sprp_07858T0:0.09697503480929868525,(Pcap_104027:0.00039687622937147700,(Psoj_109271:0.00121020870700763424,(Pram_71924:0.00857928068262667036,Pinf_13404T0:0.00172585041373604208)54:0.00395255600334274305)85:0.01522111036128112951)100:0.11825768932619719342)100:0.28528673887278227506,((Tpse_40925:0.10644175030806185545,(Ptrc_11441:0.07506879501820849787,Fcyl_174108:0.11794950793301120506)100:0.07839364851222679897)100:0.59376487689145507343,Aano_38329:0.63179451943173781103)78:0.15142383589213948003)100:0.72065559165224168936,(Bden_06102:0.32142005699400028051,Spun_03408T0:0.57402903751461631376)88:0.11066510678115838717)35:0.07891273614041052764)27:0.03345555468126028276)51:0.08332940578718193991,((Conco1_7900:0.85760178824897292937,Coere1_65120:0.67138434952192971306)80:0.11384920392452219096,(Rory_12410:0.05672322811239138840,(Pbla_39603:0.07837986635824355230,Mcir_30353:0.05376799560077138301)64:0.02729881510293826613)100:0.28841023261186704563)46:0.05264864027539016555)100:0.18293146750807601220)100:0.16327110275703143882)100:0.13063953441252706966)100:0.26075008171518604394)98:0.14029669271287234511)100:0.34496344834142717017)100:0.19618217822704298037,Snod_11456:0.12440748456807285138)93:0.03994182777931449407,Lepmu1_3609:0.07253647113767033860)100:0.04980540027102368233,(Pyrtt1_4153:0.00000094020327838334,Ptri_04610:0.00000094020327838334)100:0.04876026413229481160)56:0.00548917547856742245,Settu1_113050:0.00307470824797010040)25:0.00000094020327838334)80:0.00309037521223011480,Cocsa1_252499:0.00153338366736754310)45:0.00000094020327838334)67:0.00000094020327838334,Cocmi1_104010:0.00306754276239172086);

[tree 2: Consense majority rule phylogeny] (((((((((((((((((((Foxy_10639:100.0,Fver_09301:100.0):100.0,Fgra_03041:100.0):100.0,(((Tree_79225:100.0,TrireRUTC30_25448:100.0):100.0,((Tvir_76818:100.0,Triha1_9591:100.0):80.0,(Tatr_151043:100.0,Trias1_77086:100.0):84.0):65.0):100.0,Nhae_70495:100.0):93.0):100.0,((Valb_04315T0:100.0,Vdah_05625T0:100.0):100.0,Acral1_1062944:100.0):100.0):98.0,(((Mpoa_MAPG_02762T0:100.0,Mory_04714T0:100.0):100.0,Cprs_93824:100.0):94.0,((Pans_PODANSg804:100.0,((Spoth2_2307570:100.0,Cglo_08507:100.0):94.0,Tter_74758:100.0):98.0):100.0,((Ntet_118973:100.0,Ncra_01502:100.0):88.0,Ndis_128789:100.0):100.0):100.0):97.0):100.0,(((Bcin_09004:100.0,Sscl_00885:100.0):100.0,(Oidma1_38254:100.0,Amore1_100240:100.0):99.0):92.0,Gdes_03987T0:100.0):95.0):96.0,((((((Aspsy1_50074:100.0,Aspve1_47803:100.0):100.0,Anid_AN9120:100.0):83.0,(Anigcbs51388_3170332:100.0,Aspbr1_35001:100.0,(Acar_27118:100.0,Aspfo1_37284:100.0):62.0,Anig_R317033268:100.0):53.0,(Nfis_NFIA_114650:100.0,Afum_Afu7g01920:100.0):60.0,(Aory_AO090038000568:100.0,Afla_07897:100.0):100.0,Acla_065630:100.0,Ater_01820:100.0,Aspac1_34713:100.0):60.0,Pench1_67724:100.0):100.0,((((Cpos_05757:100.0,Cimm_RS_v2_08123:100.0):100.0,Uree_02143:100.0):100.0,(((Trub_05742T0:100.0,Aben_06116:100.0,(Tver_00102:100.0,Mgyp_07388T0:100.0):64.0):66.0,(Tton_06405T0:100.0,Tequ_07670T0:100.0):72.0):74.0,Mcan_02390T0:100.0):100.0):88.0,((Hcap_04441:100.0,Bder_04692T0:100.0):100.0,Pbra_06854T0:100.0):100.0):95.0):97.0,Eder_HMPREF1120_0453:100.0):86.0):69.0,Xanpa1_58045:100.0):57.0,(Cgra_26465:100.0,Clagr2_26465:100.0):100.0):86.0,((((((((((Cgla138_50293059:100.0,(Ndai_365984393:100.0,Scas_AAT41619:100.0):86.0,((Tpha_367005628:100.0,Vpol_186p2.t1:100.0):98.0,(Tdel_367008806:100.0,Zrou_ZYRO0D11770g:100.0):74.0):52.0,(((Smik_AAT42139:100.0,(Sbay_AAT42142:100.0,Skud_AAT42141:100.0):62.0):51.0,Scer_CAA99357:100.0):58.0,Spar_20881_YOR151C:100.0):97.0):100.0,(Klac_KLLA0B05577g:100.0,(Sklu_AAT12529:100.0,(Kthe_KLTH0G02970g:100.0,Kwal_18783:100.0):85.0):58.0,(Ecym_363752349:100.0,Egos_AFR404C:100.0):82.0):95.0):99.0,Hanva1_56526:100.0):100.0,Wican1_53842:100.0):100.0,((((Psti_34146:100.0,Spapa3_53113:100.0):85.0,((Calb_01218:100.0,Ctro_04456:100.0):100.0,(Cpar_00865:100.0,Lelo_01430:100.0):100.0):100.0):60.0,Canta1_315886:100.0,(Clus_03629:100.0,Metbi1_32161:100.0):93.0,Hypbu1_152096:100.0,Cgui_02833:100.0,Cante1_117313:100.0,Dhan_AAT12540:100.0):100.0,Babin1_159736:100.0):93.0):57.0,(((((((Cs961314_4297:100.0,Cs918151_4380:100.0):98.0,Csonorensis_1007:100.0):67.0,Cs941571_281:100.0):100.0,Canar1_194128:100.0):100.0,Hpol_50877:100.0):53.0,(Picme2_71110:100.0,Dekbr2_7243:100.0):55.0):100.0,Ppas_254567802:100.0):97.0):65.0,Ascru1_74872:100.0):99.0,(Ylip_YALI0D03718g:100.0,Nadfu1_82421:100.0):100.0):85.0,(Canca1_32658:100.0,Lipst1_73345:100.0):74.0):100.0,(((((Soyt_03939T0:100.0,Soct_02073T0:100.0):100.0,Spom_SPAC23G3_01.1:100.0):99.0,Sjap_04030:100.0):100.0,Saico1_33216:100.0):81.0,((((Mcir_30353:100.0,Pbla_39603:100.0):64.0,Rory_12410:100.0):100.0,(((((Psoj_109271:100.0,(Pram_71924:100.0,Pinf_13404T0:100.0):54.0):85.0,Pcap_104027:100.0):100.0,Sprp_07858T0:100.0):100.0,(((Ptrc_11441:100.0,Fcyl_174108:100.0):100.0,Tpse_40925:100.0):100.0,Aano_38329:100.0):78.0):100.0,(Eint_303390954:100.0,Ecun_0250_10:100.0):100.0,(Catan1_53944:100.0,Amac_10446T0:100.0):100.0,(Bden_06102:100.0,Spun_03408T0:100.0):88.0,Ganpr1_95064:100.0,PirE2_17500:100.0):51.0,(Conco1_7900:100.0,Coere1_65120:100.0):80.0):100.0,(((((Pgra_03411:100.0,Putr_03658T0:100.0):100.0,(Mlar_47615:100.0,Croqu1_75937:100.0):62.0):100.0,(Sros_22879:100.0,Rhoba1_66967:100.0):100.0):97.0,Mixos1_14551:100.0):100.0,(((((((((((Sclci1_1207496:100.0,(Pismi1_671431:100.0,Pisti1_134697:100.0):100.0):100.0,Paxin1_64693:100.0):62.0,Conpu1_116067:100.0,Hydpi2_174443:100.0):74.0,((Slac_353774:100.0,SerlaS7_491686:100.0):100.0,Serlavarsha1_40234:100.0):100.0):63.0,((Abibi_113824:100.0,Abibu_117576:100.0):100.0,Post_49642:100.0):52.0,Ccin_01820:100.0,Gymlu1_65950:100.0,Pilcr1_813588:100.0,((Hebcy1_452187:100.0,Hypsu1_182098:100.0):51.0,Galma1_234313:100.0):90.0,Lbic_ws_229137:100.0,Plicr1_171803:100.0):84.0,Scom_73589:100.0,(((((Phaca1_247866:100.0,Pchr_7545:100.0):91.0,Phlgi1_125799:100.0):91.0,Bjead1_163844:100.0):90.0,((Csub_110450:100.0,((Fompi1_100215:100.0,(Ppla_117322:100.0,PosplRSB12_1042494:100.0):100.0):89.0,Wolco1_135432:100.0):92.0):86.0,(Trave1_33021:100.0,Dicsq1_164685:100.0):100.0):51.0):83.0,Punst1_48856:100.0):59.0,(Jaaar1_121085:100.0,Glotr1_135096:100.0):98.0,(Stehi1_89162:100.0,Hann_149541:100.0):100.0,Fomme1_19006:100.0):87.0,Sphst1_215952:100.0):75.0,(Sebve1_331996:100.0,Aurde1_111514:100.0):61.0):77.0,(Botbo1_27165:100.0,Tulca1_229733:100.0):74.0):97.0,Dacsp1_23500:100.0):89.0,(Mglo_0889:100.0,Umay_AAS67525:100.0):100.0):66.0,((Cneo_H99_01235:100.0,(Cnew_AEX32502:100.0,Cneg_AEX32502:100.0):58.0,(Cneb_CNBD2790.t1:100.0,Cnej_CND03540.t1:100.0):93.0):100.0,Tmes_70991:100.0):100.0):66.0):100.0):100.0):100.0):98.0,(((Aciri1iso_149678:100.0,Bauco1_65631:100.0):100.0,((Mfij_58161:100.0,(Cerzm1_65146:100.0,Sepmu1_148144:100.0):64.0):87.0,Mgra_84586:100.0,(Dotse1_42537:100.0,Clafu1_190720:100.0):100.0):100.0):100.0,Aurpuvarsub1_6397:100.0):100.0):100.0,(Hyspu1_4193:100.0,Rhyru1_2106:100.0):100.0):100.0,Snod_11456:100.0):93.0,Lepmu1_3609:100.0):100.0,(Ptri_04610:100.0,Pyrtt1_4153:100.0):100.0):56.0,Coclu2_65013:100.0,Settu1_113050:100.0):80.0,(CocheC4_140984:100.0,Cocvi1_94880:100.0,Cocca1_101489:100.0):67.0,Cocsa1_252499:100.0):67.0,CocheC5_72858:100.0):100.0,Cocmi1_104010:100.0);

[tree 3: Curated consensus species phylogeny] (((Aano:3.0,(Tpse:2.0,(Ptrc:1.0,Fcyl:1.0):1.0):1.0):2.0,(Sprp:4.0,(Pcap:3.0,(Psoj:2.0,(Pram:1.0,Pinf:1.0):1.0):1.0):1.0):1.0):12.0,(PirE2:16.0,Ganpr1:16.0,(Eint:1.0,Ecun:1.0):15.0,(Catan1:1.0,Amac:1.0):15.0,(Bden:1.0,Spun:1.0):15.0,(Conco1:15.0,Coere1:15.0,(Pbla:2.0,(Rory:1.0,Mcir:1.0):1.0):13.0,(((Mglo:1.0,Umay:1.0):11.0,(Tmes:4.0,((Cnew:1.0,Cneg:1.0):2.0,(Cneo:2.0,(Cneb:1.0,Cnej:1.0):1.0):1.0):1.0):8.0,(Mixos1:4.0,((Sros:1.0,Rhoba1:1.0):2.0,(Mlar:2.0,Croqu1:2.0,(Pgra:1.0,Putr:1.0):1.0):1.0):1.0):8.0,(Dacsp1:11.0,((Botbo1:1.0,Tulca1:1.0):9.0,(Sebve1:9.0,Aurde1:9.0,(Sphst1:8.0,(Scom:7.0,Fomme1:7.0,Jaaar1:7.0,Glotr1:7.0,Punst1:7.0,(Stehi1:1.0,Hann:1.0):6.0,(Bjead1:3.0,(Phlgi1:2.0,(Phaca1:1.0,Pchr:1.0):1.0):1.0):4.0,((Trave1:1.0,Dicsq1:1.0):4.0,(Csub:4.0,(Wolco1:3.0,(Fompi1:2.0,(Ppla:1.0,PosplRSB12:1.0):1.0):1.0):1.0):1.0):2.0,(Pilcr1:6.0,Plicr1:6.0,(Post:5.0,((Abibi:1.0,Abibu:1.0):3.0,(Ccin:3.0,Gymlu1:3.0,Lbic:3.0,(Galma1:2.0,(Hebcy1:1.0,Hypsu1:1.0):1.0):1.0):1.0):1.0):1.0,((Serlavarsha1:2.0,(Slac:1.0,SerlaS7:1.0):1.0):3.0,(Conpu1:4.0,Hydpi2:4.0,(Paxin1:3.0,(Sclci1:2.0,(Pismi1:1.0,Pisti1:1.0):1.0):1.0):1.0):1.0):1.0):1.0):1.0):1.0):1.0):1.0):1.0):2.0,((Saico1:4.0,(Sjap:3.0,(Spom:2.0,(Soyt:1.0,Soct:1.0):1.0):1.0):1.0):9.0,((Canca1:11.0,Lipst1:11.0,(Ylip:10.0,Nadfu1:10.0,(Ascru1:9.0,((Ppas:5.0,(Picme2:4.0,Dekbr2:4.0,(Hpol:3.0,(Canar1:2.0,(Cs941571:1.0,Csonorensis:1.0,Cs961314:1.0,Cs918151:1.0):1.0):1.0):1.0):1.0):3.0,((Babin1:4.0,(Canta1:3.0,Hypbu1:3.0,Cgui:3.0,Cante1:3.0,Dhan:3.0,Psti:3.0,Spapa3:3.0,(Clus:1.0,Metbi1:1.0):2.0,((Calb:1.0,Ctro:1.0):1.0,(Cpar:1.0,Lelo:1.0):1.0):1.0):1.0):3.0,(Wican1:6.0,(Hanva1:5.0,((Klac:3.0,(Ecym:1.0,Egos:1.0):2.0,(Sklu:2.0,(Kthe:1.0,Kwal:1.0):1.0):1.0):1.0,(Cgla138:3.0,(Ndai:1.0,Scas:1.0):2.0,(Tdel:2.0,Zrou:2.0,(Tpha:1.0,Vpol:1.0):1.0):1.0,(Spar:1.0,Scer:1.0,Smik:1.0,Sbay:1.0,Skud:1.0):2.0):1.0):1.0):1.0):1.0):1.0):1.0):1.0):1.0):1.0,((Xanpa1:1.0,Clagr2:1.0):7.0,((Aurpuvarsub1:5.0,((Aciri1iso:1.0,Bauco1:1.0):3.0,(Mgra:3.0,(Dotse1:1.0,Clafu1:1.0):2.0,(Mfij:2.0,(Cerzm1:1.0,Sepmu1:1.0):1.0):1.0):1.0):1.0):1.0,((Hyspu1:1.0,Rhyru1:1.0):4.0,(Snod:4.0,(Lepmu1:3.0,((Ptri:1.0,Pyrtt1:1.0):1.0,(Coclu2:1.0,Settu1:1.0,Cocsa1:1.0,CocheC5:1.0,Cocmi1:1.0,CocheC4:1.0,Cocvi1:1.0,Cocca1:1.0):1.0):1.0):1.0):1.0):1.0):2.0,((Gdes:3.0,((Bcin:1.0,Sscl:1.0):1.0,(Oidma1:1.0,Amore1:1.0):1.0):1.0):4.0,(((Cprs:2.0,(Mpoa:1.0,Mory:1.0):1.0):2.0,((Ndis:1.0,Ntet:1.0,Ncra:1.0):2.0,(Pans:2.0,(Tter:1.0,Spoth2:1.0,Cglo:1.0):1.0):1.0):1.0):2.0,((Acral1:2.0,(Valb:1.0,Vdah:1.0):1.0):3.0,((Nhae:3.0,(Fgra:2.0,(Foxy:1.0,Fver:1.0):1.0):1.0):1.0,((Tree:1.0,TrireRUTC30:1.0):2.0,((Tvir:1.0,Triha1:1.0):1.0,(Tatr:1.0,Trias1:1.0):1.0):1.0):1.0):1.0):1.0):1.0):1.0,(Eder:7.0,(((Pbra:2.0,(Hcap:1.0,Bder:1.0):1.0):3.0,((Uree:2.0,(Cpos:1.0,Cimm:1.0):1.0):2.0,(Mcan:3.0,(Trub:2.0,Mgyp:2.0,Tver:2.0,Aben:2.0,(Tton:1.0,Tequ:1.0):1.0):1.0):1.0):1.0):1.0,(Pench1:4.0,(Acla:3.0,Ater:3.0,Aspac1:3.0,(Nfis:1.0,Afum:1.0):2.0,(Aory:1.0,Afla:1.0):2.0,(Anid:2.0,(Aspsy1:1.0,Aspve1:1.0):1.0):1.0,(Anigcbs51388:1.0,Aspbr1:1.0,Anig:1.0,Acar:1.0,Aspfo1:1.0):2.0):1.0):2.0):1.0):1.0):4.0):1.0):1.0):1.0):1.0):1.0);

[tree 4: Curated bifurcating species phylogeny] (((Aano:3.0,(Tpse:2.0,(Fcyl:1.0,Ptrc:1.0):1.0):1.0):2.0,(Sprp:4.0,(Pcap:3.0,(Psoj:2.0,(Pinf:1.0,Pram:1.0):1.0):1.0):1.0):1.0):20.0,((Spun:1.0,Bden:1.0):23.0,((Ganpr1:3.0,((Amac:1.0,Catan1:1.0):1.0,(Ecun:1.0,Eint:1.0):1.0):1.0):20.0,(PirE2:22.0,(((Coere1:1.0,Conco1:1.0):2.0,(Pbla:2.0,(Mcir:1.0,Rory:1.0):1.0):1.0):18.0,(((Tmes:4.0,((Cneg:1.0,Cnew:1.0):2.0,(Cneo:2.0,(Cnej:1.0,Cneb:1.0):1.0):1.0):1.0):15.0,((Mixos1:4.0,((Rhoba1:1.0,Sros:1.0):2.0,((Putr:1.0,Pgra:1.0):1.0,(Croqu1:1.0,Mlar:1.0):1.0):1.0):1.0):14.0,((Umay:1.0,Mglo:1.0):16.0,(Dacsp1:16.0,((Tulca1:1.0,Botbo1:1.0):14.0,((Sebve1:1.0,Aurde1:1.0):13.0,(Sphst1:13.0,(Fomme1:12.0,((Hann:1.0,Stehi1:1.0):10.0,(Scom:10.0,(((Jaaar1:1.0,Glotr1:1.0):7.0,(Punst1:7.0,((Bjead1:3.0,(Phlgi1:2.0,(Pchr:1.0,Phaca1:1.0):1.0):1.0):3.0,((Dicsq1:1.0,Trave1:1.0):4.0,(Csub:4.0,(Wolco1:3.0,(Fompi1:2.0,(PosplRSB12:1.0,Ppla:1.0):1.0):1.0):1.0):1.0):1.0):1.0):1.0):1.0,((Post:7.0,((Abibu:1.0,Abibi:1.0):5.0,(Ccin:5.0,(Lbic:4.0,(Gymlu1:3.0,(Galma1:2.0,(Hypsu1:1.0,Hebcy1:1.0):1.0):1.0):1.0):1.0):1.0):1.0):1.0,((Plicr1:1.0,Pilcr1:1.0):6.0,((Serlavarsha1:2.0,(SerlaS7:1.0,Slac:1.0):1.0):4.0,(Conpu1:5.0,(Hydpi2:4.0,(Paxin1:3.0,(Sclci1:2.0,(Pisti1:1.0,Pismi1:1.0):1.0):1.0):1.0):1.0):1.0):1.0):1.0):1.0):1.0):1.0):1.0):1.0):1.0):1.0):1.0):1.0):1.0):1.0):1.0,((Saico1:4.0,(Sjap:3.0,(Spom:2.0,(Soct:1.0,Soyt:1.0):1.0):1.0):1.0):12.0,(((Canca1:1.0,Lipst1:1.0):13.0,((Ylip:1.0,Nadfu1:1.0):12.0,(Ascru1:12.0,((Ppas:7.0,((Dekbr2:1.0,Picme2:1.0):5.0,(Hpol:5.0,(Canar1:4.0,(Cs941571:3.0,(Csonorensis:2.0,(Cs961314:1.0,Cs918151:1.0):1.0):1.0):1.0):1.0):1.0):1.0):4.0,((Babin1:8.0,(Cgui:7.0,(Canta1:6.0,((Cante1:1.0,Dhan:1.0):4.0,((Hypbu1:2.0,(Metbi1:1.0,Clus:1.0):1.0):2.0,((Spapa3:1.0,Psti:1.0):2.0,((Ctro:1.0,Calb:1.0):1.0,(Lelo:1.0,Cpar:1.0):1.0):1.0):1.0):1.0):1.0):1.0):1.0):2.0,(Wican1:9.0,(Hanva1:8.0,((Klac:4.0,((Egos:1.0,Ecym:1.0):2.0,(Sklu:2.0,(Kwal:1.0,Kthe:1.0):1.0):1.0):1.0):3.0,((Cgla138:3.0,((Vpol:1.0,Tpha:1.0):1.0,(Tdel:1.0,Zrou:1.0):1.0):1.0):3.0,((Scas:1.0,Ndai:1.0):4.0,(Spar:4.0,(Scer:3.0,(Smik:2.0,(Sbay:1.0,Skud:1.0):1.0):1.0):1.0):1.0):1.0):1.0):1.0):1.0):1.0):1.0):1.0):1.0):1.0):1.0,(((Aurpuvarsub1:6.0,((Bauco1:1.0,Aciri1iso:1.0):4.0,((Clafu1:1.0,Dotse1:1.0):3.0,(Mgra:3.0,(Mfij:2.0,(Sepmu1:1.0,Cerzm1:1.0):1.0):1.0):1.0):1.0):1.0):5.0,((Rhyru1:1.0,Hyspu1:1.0):9.0,(Snod:9.0,(Lepmu1:8.0,((Pyrtt1:1.0,Ptri:1.0):6.0,(Settu1:6.0,(Coclu2:5.0,(Cocsa1:4.0,((Cocmi1:1.0,CocheC5:1.0):2.0,(CocheC4:2.0,(Cocca1:1.0,Cocvi1:1.0):1.0):1.0):1.0):1.0):1.0):1.0):1.0):1.0):1.0):1.0):3.0,((Clagr2:1.0,Xanpa1:1.0):12.0,(((Gdes:3.0,((Sscl:1.0,Bcin:1.0):1.0,(Amore1:1.0,Oidma1:1.0):1.0):1.0):4.0,(((Cprs:2.0,(Mory:1.0,Mpoa:1.0):1.0):3.0,((Ndis:2.0,(Ntet:1.0,Ncra:1.0):1.0):2.0,(Pans:3.0,(Tter:2.0,(Spoth2:1.0,Cglo:1.0):1.0):1.0):1.0):1.0):1.0,((Acral1:2.0,(Vdah:1.0,Valb:1.0):1.0):3.0,((Nhae:3.0,(Fgra:2.0,(Fver:1.0,Foxy:1.0):1.0):1.0):1.0,((TrireRUTC30:1.0,Tree:1.0):2.0,((Triha1:1.0,Tvir:1.0):1.0,(Trias1:1.0,Tatr:1.0):1.0):1.0):1.0):1.0):1.0):1.0):5.0,(Eder:11.0,(((Pbra:2.0,(Bder:1.0,Hcap:1.0):1.0):4.0,((Uree:2.0,(Cimm:1.0,Cpos:1.0):1.0):3.0,(Mcan:4.0,((Tequ:1.0,Tton:1.0):2.0,((Trub:1.0,Aben:1.0):1.0,(Mgyp:1.0,Tver:1.0):1.0):1.0):1.0):1.0):1.0):4.0,(Pench1:9.0,(Acla:8.0,((Afum:1.0,Nfis:1.0):6.0,(Ater:6.0,((Anid:2.0,(Aspve1:1.0,Aspsy1:1.0):1.0):3.0,((Aspac1:2.0,(Afla:1.0,Aory:1.0):1.0):2.0,((Aspfo1:1.0,Acar:1.0):2.0,(Aspbr1:2.0,(Anig:1.0,Anigcbs51388:1.0):1.0):1.0):1.0):1.0):1.0):1.0):1.0):1.0):1.0):1.0):1.0):1.0):1.0):1.0):1.0):4.0):1.0):1.0):1.0):1.0):1.0);
